# Bioaccumulation of pharmaceuticals and personal care product chemicals in fish exposed to wastewater effluent in an urban wetland

**DOI:** 10.1038/s41598-017-15462-x

**Published:** 2017-12-05

**Authors:** Derek Muir, Denina Simmons, Xiaowa Wang, Tom Peart, Maria Villella, Jason Miller, Jim Sherry

**Affiliations:** Aquatic Contaminants Research Division, Water and Science Technology Directorate, Environment & Climate Change Canada, Burlington, ON L7S 1A1 Canada

## Abstract

The bioaccumulation of a broad range of pharmaceuticals and personal care product chemicals (PPCPs) was studied in Cootes Paradise Marsh (CPM), an urban wetland that receives tertiary treated municipal waste waters as well as urban storm runoff. We measured PPCPs in caged and wild goldfish, as well as wild carp, and compared observed bioaccumulation factors (BAF_P_) using concentrations in surface waters and fish blood plasma, with modeled BAFs. Thirty-two PPCPs were detected in water from the central CPM site (CPM3) while 64 PPCPs were found at higher concentrations at a site immediately downstream of the effluent outflow (CPM1). Following a 3-week deployment, 15 PPCPs were detected in the plasma of caged goldfish at CPM1, and 14 at CPM3, compared to only 3 in goldfish caged at a reference site. The highest BAF_P_ in goldfish were for the antidepressant Σfluoxetine averaging 386 L/kg in caged and 906 L/kg in wild goldfish, respectively. In carp, ΣDiazepam (diazepam and oxazepam) had the highest BAF_P_ (927 L/kg). This study identified a broader range of PPCPs in fish and surface waters than previously reported. However, modeled BAFs did not show good agreement with observed whole body or plasma BAFs, demonstrating that more work is needed to better explain bioaccumulation of PPCPs.

## Introduction

Pharmaceuticals and personal care products (PPCPs) and endocrine disrupting compounds (EDCs) have been measured in municipal wastewater effluents and in receiving waters in southern Ontario (Canada) including in Hamilton Harbour^1–5^ and the adjoining Cootes Paradise Marsh (CPM)^6,7^. Chemicals in some personal care products such as musks, triclosan, UV filters, and alkyl phenols have been widely determined in wild fish^8,9^ including in fish from rivers and harbours in the North American Great Lakes region^10–14^. Many of these latter compounds are relatively hydrophobic and predicted to bioaccumulate in fish^15^. However, there have been fewer measurements of pharmaceuticals in wild fish possibly because these substances are more polar and assumed to be less bioaccumulative and more readily metabolised^11,16,17^. However, Howard and Muir^18^ evaluated the pharmaceuticals in active production/use and concluded that about 20% of the high production volume active ingredients, that had not yet been detected in environmental media, were potentially persistent and bioaccumulative in fish. Chu and Metcalfe^19^ detected the antidepressants paroxetine and fluoxetine as well as the active metabolite, norfluoxetine, in fish from Hamilton Harbour using whole fish tissue. Huerta *et al*.^9^ reviewed the literature on measurements of pharmaceuticals in tissues of wild fish to 2012 and found about 40 compounds had been reported. Since then larger numbers have been measured, in either whole fish or muscle^20–24^ and in various other tissues including plasma^25–28^. Nevertheless the number analysed to date is far smaller than the number of active pharmaceutical ingredients^9^. Given that pharmaceuticals are biologically active chemicals, further exposure information, particularly for internal tissue doses, would be useful so that the risks to fish are better understood^29–31^.

Our objective was to measure PPCPs in caged and wild goldfish, as well as in wild carp, from Cootes Paradise Marsh (CPM), in addition to a reference site (Jordan Harbour, JH), in order to help interpret proteomic, metabolomic, and behavioral effects data being generated in companion studies on the same fish^32,33^. CPM is a 250-ha wetland, within the 850 ha conservation area owned and managed by the Royal Botanical Gardens and is located at the western tip of Hamilton Harbour (Supplementary Information Figure S1). It receives water from 3 urban creeks as well as a wastewater treatment plant which uses tertiary treatment. The nutrient and organic contaminant inputs and distribution in the marsh are described in detail by Kelton *et al*.^34^ and Mayer *et al*.^7^.

Measurement of pharmaceuticals in fish has been proposed as a way of assessing potential hazards by comparing observed levels in fish blood plasma to human therapeutic plasma doses^35^. An increasing number of studies have included measurements of pharmaceuticals in fish plasma^25,26,36,37^. Therefore plasma was chosen as the matrix for PPCP analysis. We were also interested to determine bioaccumulation factors for PPCPs in fish by comparing concentrations in waters and in fish plasma from the same sites. Previous studies of bioaccumulation of pharmaceuticals in fish tissues have generally targeted limited numbers of compounds^9,16,38,39^, however the recent availability of methodology for quantitative analysis of over 120 analytes has enabled broader suites to be determined in invertebrates and fishes^20,40^. By addressing a larger suite of PPCPs than has previously been determined and doing so in fish plasma we hoped to help to prioritize which compounds represent the greatest exposure risk for wild fish near municipal wastewater outfalls.

## Results and Discussion

### PPCPs in water

**Figure 1:**
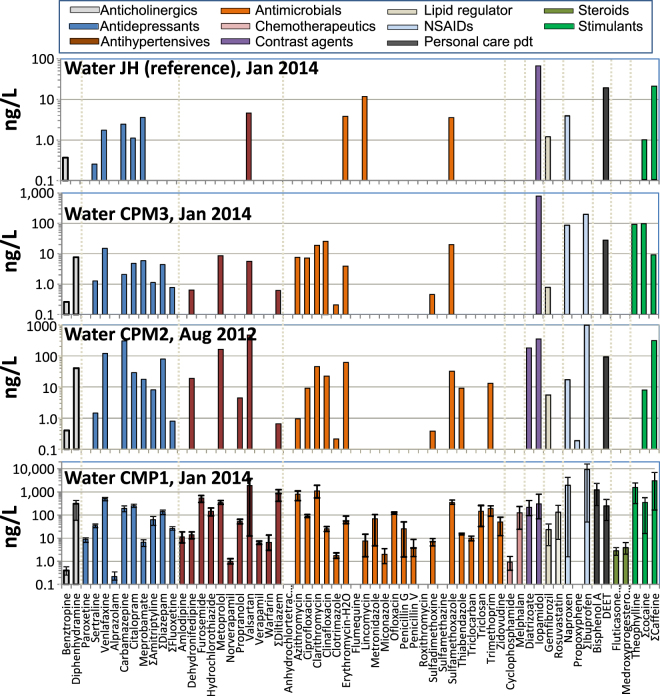
PPCPs detected in surface waters of Cootes Paradise Marsh. Inflowing WWTP influenced waters of the Desjardins Canal outflow CPM1, middle and distant sites CPM2, CPM3, and in Jordan Harbour (JH), a reference site. Results for CPM1 are averages of 4 sampling times (ranges shown as vertical bars). CPM2 and CPM3 are single samples while JH is an average of two samples. The horizontal axis labels are for 62 analytes (including 7 with combined parent and transformation products) detected in water at CPM1 (full tabulated list in SI Table S2).

A large number of compounds (64/127) at relatively high concentrations were found at CPM1, at the outlet of the Desjardins Canal, which contains mainly the tertiary treated effluent of the Dundas Wastewater Treatment Plant (Fig. 1; SI Table S2). At site CPM2, which is about 450 m downstream of CPM1, the number of PPCPs detected was lower (39/127) than at CPM1, indicating degradation and dilution was occurring. Thirty-two of 127 PPCP target analytes were detected in water from the central CPM site (CPM3) in samples collected in January and July 2014 (SI Table S2; Fig. 1). PPCPs were detected at the same frequency and generally at a similar range of concentrations in samples collected at CPM3 in August 2012. This latter sample had been stored at −20 °C until analysed in 2014 while the January and July sample holding times (4 °C) were approximately 40 and 190 days, respectively. Thus despite different holding times, samples from three different time periods had the same PPCP detection frequency and range of concentrations. The effect of holding times is discussed further in Supplementary Information Detection frequency was considerably lower at the reference site (JH), with 17 and 16 of 127 analytes detected in January and July, respectively (SI Table S2).

Considering all sampling times and sites, altogether a total of 69 individual analytes (62 with transformation products combined with parents) were detectable in water and fish plasma. The major PPCPs present in Desjardins Canal outflowing waters with concentrations in the range of 1000 to 11800 ng/L were ibuprofen (including its hydroxyl degradation product), caffeine (and its degradation product 1,7-dimethylxanthine), naproxen, valsartan, theophylline, azithromycin and bisphenol A. Figure 1 compares the concentration profiles of the detected PPCPs in January 2014 from the Desjardins Canal outflow (CPM1), at CPM2, CPM3, and JH, the reference site. With the exception of iopamidol (a contrast agent), which had higher concentrations at CPM3 in January sampling, concentrations were 1.3 to 12000-times higher closer to the wastewater outfall (CPM1) compared to the central marsh site (CPM3). Concentrations of iopamidol were also higher at CPM3 compared to the CPM1 in July 2014 (SI Table S2). The source of iopamidol is therefore unlikely to be the WWTP effluent. CPM3 waters are influenced by inflows from 3 streams (Borer’s Creek, Chedoke Creek, Spencer’s Creek). The Chedoke Creek catchment is a possible source because it is heavily urbanized and the stream consists mainly of underground channels as well as a combined sewer overflows^7,34^. Concentrations of PPCPs in JH were generally much lower than at CPM3, however, several compounds including iopamidol, as well as antidepressants diazepam and meprobamate, the lipid regulator gemfibrozil and the antibiotic lincomycin were present at similar concentrations as those at CPM3 (Fig. 1). The source of these compounds for JH is probably leakage from domestic septic systems within the catchment of Twenty Mile Creek, the main inflowing stream; municipal wastewater from the town of Jordan does not enter the harbour^41^.

Concentrations of PPCPs in surface waters were generally higher in January than in July at CPM1, CPM2 and JH (SI Figure S1). Higher water temperatures in July (20–22 °C) than in January (0–5 °C), as well as greater sunlight irradiation of the surface waters in summer, likely led to more rapid transformation^42^. Seasonal differences in use of pharmaceuticals could also be important. The longer storage time for the July 2014 samples could also have led to lower measured concentrations as discussed further in the Supplementary Information. A relatively high day to day variation of PPCP concentrations was observed in the 4 sampling times from January 21 to February 3 at CPM1 (SI Table S1). For example iopamidol increased steadily from 68 to 783 ng/L over this period while others (penicillin G, melphalan, diltiazem, and ibuprofen) declined. Overall about 40% of the 63 compounds had relative standard deviations of >50% in the January sampling at CPM1. This variation was not due to changes in water flow. Detailed basic water chemistry parameters are given in SI Table S6. Basic water chemistry parameters remained relatively constant over the January sampling period at CPM 1 (Canal outlet) and CPM3 (SI Table S3).

### PPCPs in fish plasma

**Figure 2:**
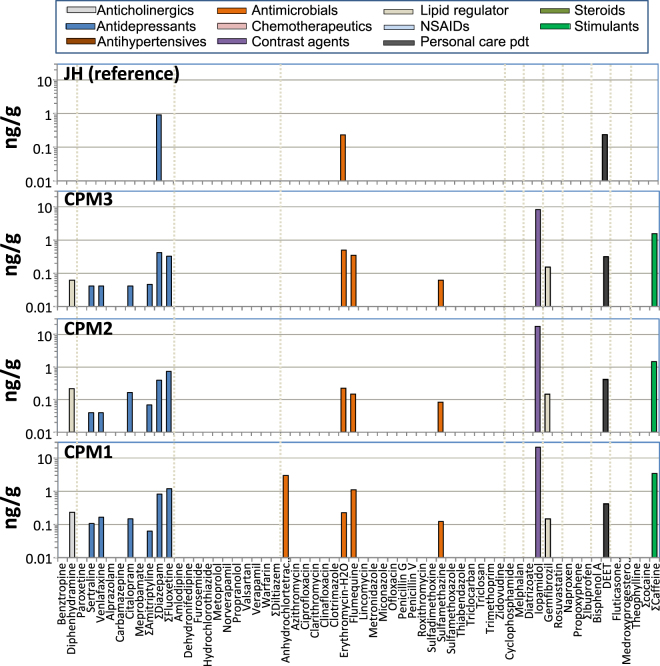
PPCPs detected in plasma of caged goldfish at the same sampling locations in Cootes Paradise Marsh (July 2014). Bars represent concentrations in a single pooled sample from 25 fish. Cage deployment sites are the same as those for water. See SI Table S1 for a tabulated list of the analytes on the horizontal axis.

The detection frequency of the PPCPs in pooled plasma of caged goldfish was much lower than in surface waters collected during the 3 week deployment period (Fig. 2) with only 16 of 127 analytes detectable at CPM1, and 15 at CPM2 and CPM3 (SI Table S4). The compounds detected were the same at all 3 CPM sites except that anhydrochlortetracyclin, a chlorotetracycline analog, was detected at CPM1 but not in samples from CPM2 and CPM3 (SI Table S4). Nine of the 16 PPCPs were antidepressants; sertraline, venlafaxine, citalopram, ΣAmitriptyline (amitriptyline +10-hydroxy-amitriptyline, ΣDiazepam (diazepam + oxazepam) and ΣFluoxetine (fluoxetine + norfluoxetine). Concentrations in goldfish plasma were 1.3 to 4-fold higher in the caged goldfish at the site with high wastewater exposure (CPM1) compared to fish found at the far-field site in the marsh (CPM3) with two exceptions; the antibiotic erythromycin was two-fold higher at CPM3 (0.50 ng/g vs 0.22 ng/g), and the lipid regulator gemfibrozil, which showed no difference in concentration (0.15 ng/g; Fig. 2). Goldfish caged at JH had a much lower frequency of detection with just 3 of 127 analytes detected (oxazepam, erythromycin and DEET). Plasma concentrations of oxazepam (0.91 ng/g) and erythromycin (0.23 ng/g) in the JH goldfish were within 10% to those at CPM1 (SI Table S4), despite this reference site not having the direct influence of municipal wastewater.

**Figure 3:**
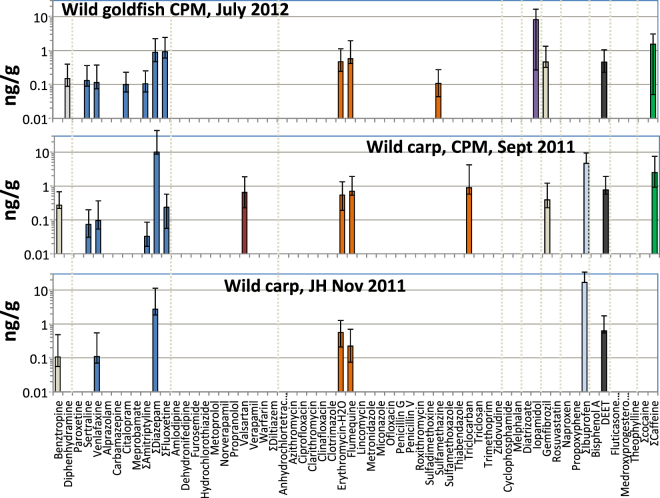
Mean concentrations and range of PPCPs detected in plasma of wild goldfish and wild carp collected in Cootes Paradise Marsh and the reference site (JH). Error bars represent standard deviations. See SI Table S1 for a tabulated list of the analytes on the horizontal axis and SI Table S4 and S5 for concentrations in individual sample pools.

The detection frequency of PPCPs in wild goldfish collected from the CPM in 2012 was nearly identical to that of caged fish at CPM3 in 2014 with only caffeine undetectable in the wild fish (Fig. 3). However, concentrations of the 16 analytes were 1.5 to 4.1-fold higher in the wild fish (SI Table S4) except for erythromycin and iopamidol which were present at nearly identical concentrations in caged and wild goldfish. The antibiotics flumequine and sulfamethazine were detectable in caged and wild goldfish but were not detectable in central CPM surface waters in either January or July (SI Table S2). Norfluoxetine, a transformation product of fluoxetine, was also not detectable in water at CPM3 but was present in goldfish plasma.

Plasma from wild carp sampled in CPM had a smaller number of analytes detected (13/127) compared to wild goldfish (Fig. 3) and the identities of detectable compounds also differed. There were fewer antidepressants in carp (2) compared to goldfish (5), while benztropine, valsartan and triclocarban were detected in carp (SI Table S5) but not goldfish (SI Table S4). PPCP concentrations in the plasma of both species differed by less than 2-fold, except for norfluoxetine and oxazepam. Norfluoxetine was 2.9 fold higher in goldfish than in carp while oxazepam, which is a metabolite of diazepam and several other benzodiazepines, as well as a registered pharmaceutical, was 11-fold higher in carp. These differences for fish exposed to the same mixture of PPCPs probably reflect differences in metabolic capacity between carp and goldfish although the sampling for carp was one year earlier (2011 vs 2012) and therefore differences in exposure may also be influenced by temporal changes in prevailing water concentrations. Carp from JH also had a different set of PPCPs compared to caged goldfish (SI Figure S3) with benzotropine and flumequine detected in carp but not goldfish. Concentrations of erythromycin and DEET were about 2-fold higher in carp plasma than in goldfish. Brown bullhead plasma from Jordan Harbour had a low PPCP detection frequency (3/127) (SI Table S5).

### Comparison with other studies of PPCPs in water and fish

The average concentrations of PPCPs at the central marsh site (furthest from the WWTP outfall, CPM3) were generally lower than the average of mean values from other studies on pharmaceuticals in global rivers and streams (SI Table S6) summarized in the review by aus der Beek *et al*.^43^. The exceptions were for clinafloxacin, iopamidol, and theophylline which were higher than average global concentrations and clarithromycin and naproxen, which had maximum concentrations at CPM3 that exceeded average global concentrations. As noted previously, central CPM waters are influenced by inflows from 3 streams with urban sources (e.g. from combined sewer overflows) to the south, suburban development to the west, and forested parkland to the north, as well as by the Desjardin Canal WWTP inflow (Figure S1). Thus it is similar to other effluent-dominated rivers and streams that have been studied extensively in the USA, Western Europe, Japan, and China for simultaneous measurements of PPCPs in water and fish tissues^8,22,24–27,44^.

While over 600 pharmaceuticals were identified in aquatic matrices (not including biota) in the review by aus der Beek *et al*.^43^, until recently only a limited number of measurements have been made with fish plasma or indeed in tissues of wild fish in general^9^. Meador *et al*.^20^ substantially increased the detected number of pharmaceuticals detected in fish when they analysed for about 74 PPCPs and found 31 in sculpin (*Leptocottus armatus*) and 23 in juvenile chinook salmon (*Oncorhynchus tshawytscha*) (in whole body composites). Meador *et al*. used the same analytical lab as this study and a similar analytical list; a side by side comparison of the results is given in SI Table S7. Analytes present at higher concentrations in goldfish and carp plasma (amitriptyline, caffeine, DEET, diphenhydramine, erythromycin, fluoxetine/norfluoxetine, gemfibrozil and sertraline) also were prominent in salmon and sculpin tissue (SI Table S7). Other studies that have determined pharmaceuticals in fish tissues (muscle, brain or liver) and results for the compounds detected in this study are summarized in SI Table S8. Similar to the results from the study by Meador *et al*.^20^, diphenhydramine, fluoxetine, gemfibrozil, and sertraline have been widely reported in fish tissues^9^. Approximately 50 pharmaceuticals have been targeted for analysis either in plasma of wild fish^26,27,44^ or in plasma of fish exposed to final municipal wastewater effluent in the lab^36,37^. Fick *et al*.^37^ showed that rainbow trout exposed in the lab to WWTP effluent had pharmaceuticals in plasma at concentrations similar to human therapeutic doses and also demonstrated the suitability of plasma as a matrix for pharmaceuticals with a wide range of hydrophobicity and pKa. In laboratory bioconcentration tests, fish plasma has also been shown to generally have similar or higher concentrations of pharmaceuticals and their metabolites compared to muscle^45–47^. Tanoue *et al*.^25^ determined four PPCPs in common with this study (flumequine, diphenhydramine, triclocarban and sertraline) in plasma and other tissues of carp from wastewater-impacted streams and reported a similar range of concentrations in plasma (SI Table S8). Zhao *et al*.^27^ determined 9 antibiotics in fish plasma including two (sulfamethazine and erythromycin) that were included in the present study. However, concentrations were about 50-fold higher (SI Table S8).

### Bioaccumulation factors

**Figure 4:**
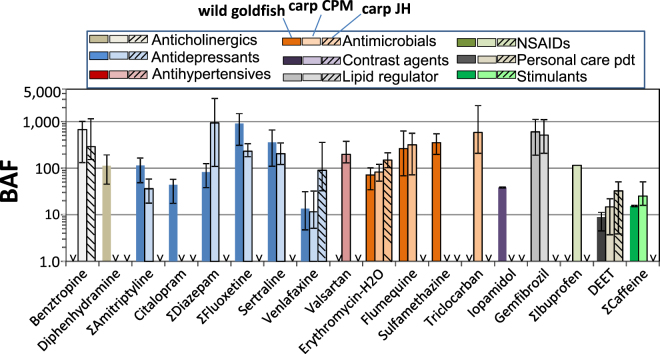
Bioaccumulation factors (L/kg) for PPCPs goldfish and carp in Cootes Paradise (conc’n in fish plasma ÷ conc’n in water) and in carp from Jordan Harbour (JH). Error bars represent standard deviation based on concentrations in fish. Substances labelled with “<” had BAF_P_ that could not be calculated due to non-detect concentrations in water or fish plasma.

The highest plasma BAF (BAF_P_) in goldfish was found for the antidepressant ΣFluoxetine, ranging from 906 L/kg in wild goldfish, and from 207–689 in caged goldfish (Fig. 4, SI Table S9). In carp, ΣDiazepam had the highest BAF_P_ (927 L/kg) mainly due to its metabolite oxazepam (Fig. 4, SI Table S9). Three compounds, carbamazepine, metoprolol and sulfamethoxazole, which were consistently detected in water from sites CPM1–3, and have been reported in other studies^43,48^, were not detected in fish plasma suggesting rapid biotransformation. BAF_P_s for the PPCPs detected in caged goldfish varied among locations in CPM (SI Table S9; Figure S4) with highest values generally at CPM3. It should be noted, however, that the BAF_P_ for goldfish at CPM2 was based on water concentrations from a sample taken in August 2012 while all others are from the deployment period in July 2014, and are therefore less certain. Combining results for caged goldfish from all 3 sites showed that BAF_P_s declined significantly (R^2^ = 0.51; P < 0.001) with increasing water concentrations (Figure S5). Liu *et al*.^49^ also reported a significant inverse relationship between the brain and liver BAFs of several antibiotics in fish and the log concentration in water. Our results for caged goldfish with identical exposure period, size and sex confirm Liu *et al*.’s observations. The lower BAF_P_ with higher water concentrations could be due to a number of factors including lower bioavailability of PPCPs at sites closer to the effluent as well as physiological impacts on the fish. In the present study pH was lower (0.4 units) and dissolved and particulate organic carbon 2-fold higher near CPM 1 and 2 (Canal outlet) versus the central marsh (CPM3) during the caging study (SI Table S3), which may have reduced uptake of many PPCPs across the gills. In a companion study using targeted metabolomics and untargeted proteomics, Simmons *et al*.^33^ found an increase in molecules in the caged goldfish at CPM1 compared to CPM2 and 3 that were related to liver necrosis. This implies decreased capacity of the more highly exposed fish to metabolize some PPCPs, which likely would reduce clearance and increase BAFs. Additionally, CYP450 phase-I metabolism by the goldfish may have been reduced by other PPCPs present in the water and detected in plasma, notably gemfibrozil, erythromycin, and fluoxetine^50,51^. Thus it is more likely that water chemistry affected uptake at the gills and was the primary factor causing lower BAFs, rather than metabolism by the fish at CPM1.

Bioaccumulation of PPCPs is influenced by pH, presence of surfactants, as well as suspended solids and thus direct measurements of exposure are needed^30,36^. Data to support exposure modelling of PPCPs and prioritization for monitoring are needed because the large number of PPCPs potentially present in effluents (as parent compounds and transformation products) represent a formidable challenge to measure individually^31,52^. With that in mind, we have used the results for water and fish plasma to evaluate two widely used modeling approaches for predicting organic contaminant bioaccumulation, the BCF-BAF model for whole body BAFs^53^ (BAF_WB_) and the fish-plasma concentration calculation approach of Berninger *et al*.^54^.

BAF_WB_s for the 18 PPCPs detectable in wild goldfish and/or carp were predicted for lower trophic level fish using the BCF-BAF model in EPISuite^53^. This model uses a predicted biotransformation rate^55^ and is therefore appropriate for screening of these substances which are expected to be accumulated from both water and food as well as undergo biotransformation by fish. Measured BAF_P_ versus predicted BAF_WB_ for PPCPs detectable in wild goldfish and carp are presented in SI Figure S6A and in SI Table S9. Measured log BAF_P_ was correlated (P < 0.05) with predicted log BAF_WB_ (r^2^ = 0.32, P = 0.044, N = 13) for goldfish when sertraline was omitted from the regression but not for carp (r^2^ = 0.18, P = 0.150, N = 13). Sertraline, the most hydrophobic compound (log octanol-water partition coefficient, log Kow = 5.29), was predicted to have a larger biotransformation half-life (t1/2) than the other compounds (35 days for a 100 g fish, versus 0.004 to 3 days for others in SI Table S9). Close inspection of the BAFs suggested that the BCF-BAF model gave predicted values within 3-fold of measured BAF_P_ for ΣDiazapam, DEET, ΣFluoxetine, and gemfibrozil in goldfish and benztropine, DEET, ΣFluoxetine, valsartan, and triclocarban in carp. These substances have log Kows ranging from 2.2–4.9 and biotransformation t1/2 s of 0.16 to 3 days (i.e. a relatively narrow range compared to the group as a whole). The BCF-BAF model predictions are based on whole fish while the measured BAF_P_s are based on plasma. As noted above, plasma and muscle concentrations are similar for many PPCPs in both wild fish and in lab bioconcentration studies although liver typically has higher concentrations^25,27,49^. Thus, BAF_WB_s would be expected to be higher. Tanoue *et al*.^25^ also found that BAF_WB_s of 15 PPCP in carp, predicted using log Kow, were much higher (1–5 orders of magnitude) compared to measured BAF_P_s.

Measured BAFs in wild fish based on plasma have been reported for only a few of the PPCPs that we detected. Zhao *et al*.^27^ reported log BAF_P_s for erythromycin-H2O and sulfamethazine ranging from 0.37–4.19 and 1.92–4.18, respectively, in tilapia and carp species in southern China. They found BAF_P_s in plasma were similar to those for bile and liver in the same fish. The plasma BAFs for erythromycin-H2O and sulfamethazine goldfish and carp in this study (log BAF_P_s 1.53–2.73) were at the lower end of the range found by Zhao *et al*.^27^. BAF_P_s in carp plasma reported by Tanoue *et al*.^25^ for 4 PPCPs in common with the present study (medians: DEET (<6.2),diphenhydramine (1.5), triclocarban (6.9) and sertraline (8.9)) were generally lower than found in the present study (SI Table S9). While plasma concentrations were similar (SI Table S8) water concentrations in their waste water impacted streams where higher compared to our central marsh (CPM3) site (eg diphenhydramine ~200x; sertraline ~20x). Du *et al*.^28^ observed a log BAF_P_ of 2.2 ± 2.0 for diphenhydramine in longear sunfish (*Lepomis megalotis*) in a wastewater influenced stream, which is within the range found in goldfish in the present study. Fick *et al*.^37^ calculated a BAF_P_ of 138–240 for sertraline in rainbow trout (*Oncorhynchus mykiss*) exposed to treated WWTP effluent from two Swedish cities. Those BAF_P_s were at the low end of the range in this study (goldfish and carp BAF_P_s of 109–659). Fick *et al*.^37^ also found much lower BAF_P_s for ibuprofen (21–58) and oxazepam (diazepam) (0.7–3.6) than observed for goldfish and carp in this study (SI Table S9).

### Fish blood-water partitioning

Bioaccumulation was also assessed by calculating fish blood-water partition coefficients (P_BW_). These were estimated using the pKa value to adjust log Kow to a log D^54^ taking into account the pH of 7.7 of the central CPM waters (Table 1). Several of the detected pharmaceuticals (erythromycin-H2O, diphenhydramine, venlafaxine, sertraline) had basic or acidic pKa’s within about 2 log units of the prevailing pH which resulted in log D having lower values than Log Kow (Table 1). Predicted P_BW_s fell within a narrower range of values (SI Figure S6B) than predicted BAFs but were not correlated with measured BAF_P_s. P_BW_ values for DEET, sertraline, and triclocarban in carp as well as citalopram and DEET in goldfish were within a factor 3 of observed BAF_P_s. However, measured BAF_P_s were under-predicted by over 100-fold for flumequine, sulfamethazine, gemfibrozil and ΣFluoxetine, while ΣAmitriptyline was over-predicted, in both species (SI Figure S6). Similarly predicted plasma concentrations (P_BW_ x water conc (ng/mL)) were within a factor 5 of measured values for DEET, sertraline, venlafaxine and ΣDiazepam; ΣAmitriptyline was over predicted while all others were under predicted (SI Figure S7). Tanoue *et al*.^25^ also compared measured BAF_P_ and predicted plasma BAFs for 15 PPCPs using log D values and the same fish-plasma concentration model^35,54^ used in the present study. Their predicted BAF_P_s were generally larger than measured BAF_P_ , with about half of the compounds within 1 order of magnitude of measured values.

**Table 1:** Range of concentrations of PPCPs detected in blood plasma of goldfish and carp along with predicted blood-water partitioning (P_BW_) and plasma concentrations (FPC).

		caged goldfish plasma	wild goldfish	Cootes carp	log D (pH 7.7)	Predicted plasma bioaccumulation parameters		Therapeutic^c^ “normal” ng/mL	toxic dose^d^ ng/mL	Cmax ng/mL^e^
		ng/g^b^	ng/g	ng/g		P_BW_	FPC ng/mL	Schulz *et al*.^56^	Schulz *et al*.^56^	Berninger & Brooks^60^
Erythromycin-H2O	Antibiotic	0.23–0.66	0.22–0.5	0.35–0.79	2.93	22.9	0.150	500–6000	12000–15000	
Sulfamethazine	Antibiotic	0.06–0.17	—	—	0.19	1.06	0.0003			
Flumequine	Antibiotic	0.16–1.37	0.15–1.1	0.16–1.23	−0.40	0.92	0.002			
Triclocarban	Antimicrobial	—	—	0.32–3.35	4.90	605	0.909			
Valsartan	Antihypertensive	—	—	0.42–1.23	−0.49	0.91	0.003	~800–6000		
Diphenhydramine	Anticholinergic	0.06–0.25	0.06–0.23	—	2.17	7.02	0.009	50–100	1000–2000	50
Benztropine	Anticholinergic	—	—	0.05–0.41	1.48	2.76	0.001	10–180	50	
Iopamidol	Contrast agent	7.85–8.35	8.2–20.9	—	−2.42	0.84	0.178			
ΣCaffeine	Stimulant	1.47–1.57	1.46–3.38	1.58–5.05	−0.07	0.98	0.097	4000–10000	15000–20000	
DEET	Repellent	0.23–0.58	0.31–0.42	0.19–1.15	2.18	7.08	0.369			
Gemfibrozil	Lipid regulator	0.15–0.86	0.15–0.15	0.16–0.83	1.83	4.30	0.003	~25000		
ΣIbuprofen	NSAID	—	—	4.67–4.67	0.68	1.34	0.054	15000–30000	200000	
ΣDiazepam	Antidepressant	0.41–1.34	0.39–0.81	1.18–33.7	2.82	19.2	0.207	100–2000	3000–5000	
Venlafaxine	Antidepressant	0.04–0.26	0.04–0.17	0.04–0.27	1.70	3.62	0.030	100–400	1000–1500	
Citalopram	Antidepressant	0.04–0.13	0.04–0.16	—	3.28	86.8	0.194	50–110	220	
ΣFluoxetine	Antidepressant	0.31–1.5	0.32–1.18	0.18–0.34	1.42	2.58	0.002	120–500	1000	330
ΣAmitriptyline	Antidepressant	0.04–0.15	0.05–0.07	0.02–0.05	4.92	626	0.566	50–300	500–600	
Sertraline	Antidepressant	0.04–0.24	0.04–0.11	0.04–0.13	3.51	59.5	0.021	50–250	290	290
Σantidepressants		0.89–3.61	0.87–2.49	1.46–34.5						

The range of human therapeutic and toxic doses is presented where available^a^.

^a^Note that results from Schultz *et al*. and Berninger & Brooks are converted from µg/mL in the original publications to ng/mL, for comparison with the fish plasma data.

^b^Concentrations are expressed as ng/g plasma as reported by the analytical laboratory but are equivalent to ng/mL assuming a density of 1 g/mL.

^c^Therapeutic: blood-plasma/serum concentrations (in general, trough at steady state) observed following therapeutically effective doses; no or only minimal side effects (drugs); “normal”: concentrations associated with no or only minimal toxic effects (other xenobiotics)^56^.

^d^Toxic: blood-plasma/serum concentrations which produce toxicity/clinically relevant side effects/symptoms.

^e^Cmax = human therapeutic dose.

Table 1 compares the blood plasma levels of PPCPs in goldfish and carp and concentrations associated with human therapeutic and toxic doses reported by Schultz *et al*.^56^. All concentrations of individual PPCPs were lower by 50-fold or more than the range of concentrations of normal human therapeutic plasma (HTP) concentrations. However, the range of concentrations of the sum of all antidepressants concentrations in carp (1.46–34.5 ng/mL) was within 1.5x of the low end of the range of HTP concentrations for several antidepressants (Table 1).

## Conclusions

This study has identified a broader range of PPCPs in fish and surface waters downstream of urban waste water treatment facilities than have been reported in most previous studies of pharmaceuticals. The major compounds detected (amitriptyline, caffeine, DEET, diphenhydramine, erythromycin, fluoxetine/norfluoxetine, gemfibrozil and sertraline) were also predominant in the study by Meador *et al*.^20^ (SI Table S7) who used the same analytical methodology and analysed whole fish homogenates. As summarized in SI Table S8, many of these substances have been detected previously in fish in other studies although less frequently in fish plasma. The data for PPCPs detected in water but not in caged or wild fish plasma, as well as comparison of goldfish versus carp, is also of interest and could be investigated further to determine whether there are molecular features that make these PPCPs more readily transformed. In particular, we suspect that the presence of gemfibrozil, erythromycin, and sulfamethazine may have inhibited cytochrome P450 (CYP450) phase-I metabolism^50,51^. This is discussed further in a companion manuscript^33^.

Although non-detectable in plasma, other PPCPs may have been detectable in liver which has been shown to have higher concentrations of some pharmaceuticals than plasma or muscle. Nevertheless, analysis of plasma provided data which could be compared directly with HTP concentrations as well as with companion studies on biological effects in caged goldfish from CPM^32,33^. BAF_WB_s estimated using the BCF-BAF model did not show good agreement with observed BAF_P_s even when including biotransformation, illustrating that models that are driven by hydrophobicity are not effective for predicting moderately polar compounds many of which are ionisable at ambient pH. On the other hand the BCF-BAF model is designed to predict BCF and BAF data for whole fish rather than plasma. Modelled P_BW_ and predicted fish plasma concentrations using log D values also did not show good agreement with measured values except for several neutral PPCPs eg triclocarban. However, this approach assumed a simple partitioning between water and plasma based on a log Kow adjusted for ionized species. More complex models, taking into account acidification of the gill surface^57^ may be needed to better explain bioaccumulation of the ionisable PPCPs.

## Methods

### Caging and sample collection

The caged goldfish (*Carassius auratus*) were deployed in CPM for 21 days from June 25/26 – July 16/17 2014. Cages were visited weekly, and fish (13/cage) were fed during the visits (20 g/cage). All animal experiments were in accordance with CCAC guidance and approved by the GLLFAS-WSTD Animal Care Committee (Government of Canada). Further details on the caging and preparation of the fish and animal care protocols are provided in Simmons *et al*.^32^. Cages were placed in 3 locations representing a gradient of exposure along the plume of the Dundas waste water treatment plant (WWTP) outfall, in the Desjardins Canal (530 m from the WWTP outfall), West Pond (975 m from the WWTP outfall), and McMaster Landing (3850 m from the outfall). A fourth site, Jordan Harbour (JH), a widening of 20 Mile Creek adjacent to Lake Ontario near Jordan ON, served as a reference site. Mass and fork length were measured and recorded. Plasma collection and storage is described in the SI. Plasma of caged goldfish was divided for proteome, metabolome and PPCP analysis. Due to limited volumes, pooled samples of plasma from 25 individual fish (50 µL/individual) was pooled to yield one sample from at each location for PPCP analysis.

Wild fish collections: Goldfish were collected in September 2012 in CPM central marsh using electro fishing and seine nets. Plasma from 9 individual male fish (500 µL/individual) was pooled to yield 3 samples for PPCP analysis. Wild carp (*Cyprinus carpio*) were collected in September 2011 in CPM and in November 2011 from JH. Plasma from 6 individual carp (2.3–3.5 g) from each location was selected for PPCP analysis. A single sample of plasma from a Brown Bullhead (*Ameiurus nebulosus*) collected in JH (April 2013) was also included for a comparison of the PPCP profile among species. Plasma samples were frozen (−80 °C) until analysis.

Water samples (1 L unfiltered; collected by filling bottles at about 10 cm below the surface) were collected from the CPM and JH in late January/early February 2014 and again in July 2014 coinciding with the goldfish caging study. Samples (N = 4) were collected at the mouth of the Desjardin Canal (CPM1) which includes the effluent from the Dundas municipal WWTP in January-February (N = 4) and in July 2014 (N = 1). Collections in the center of the marsh (site CPM3) were made in January and July 2014. Water samples were also collected in August 2012 from CPM3 and site CPM2 (See SI Information Figure S1 and Table S1). In JH, the water sampling was near the outlet to Lake Ontario (SI Figure S2). Samples collected in August 2012 were frozen (−20 °C) and then defrosted before being shipped to the analytical lab. All samples collected in 2014 were held at 4 °C in the dark until analysed. Samples collected in January 2014 were analysed within 40 days of collection while samples collected in July 2014 were analysed in February 2015, approximately 190 days after collection.

A separate set of water samples was collected at the same time for determination of particulate organic carbon (POC) and dissolved organic carbon (DOC) as well as other basic water chemistry parameters (SI Table S3). Water pH, conductivity, temperature and dissolved oxygen were measured during July field sampling using a YSI multimeter (YSI Inc., Yellow Springs, OH). All samples were kept at 4 °C and in the dark prior to analysis by the National Laboratory for Environmental Testing (Environment Canada, Burlington, ON) following standard protocols^58^.

### PPCP analysis of water and plasma

A total of 20 fish plasma samples from the pooled caged fish (7) and the wild fish (13) along with 12 water samples were selected for analysis. Samples were analysed by SGS AXYS Analytical Services (Sidney BC) using their method MLA-075 (version 6)^59^ which is a modification of US EPA Method 1694^38^. This method targeted 127 PPCPs including antibiotics, beta-blockers, antidepressants, anti-inflammatories, anticonvulsants, and personal care products such as the insect repellent, N,N-Diethyl-m-toluamide (DEET), and the antimicrobial, triclocarban. A full list of analytes is provided in Supplementary Information. Mass labelled^13^C-, or deuterated surrogate internal standards were used for quantification of all analytes via the isotope dilution/surrogate standard quantitation method. Prior to extraction and/or clean-up procedures, aqueous samples were adjusted to pH 2.0, spiked with extraction surrogate standards, then adjusted to pH 4 using tetrasodium ethylenediamine-tetraacetate dihydrate. Plasma samples (2.3–2.5 g) were spiked with extraction surrogate standards, sonicated with 20 mL acetonitrile, then diluted with ultra-pure water to 200 mL. The extracts were cleaned up by solid phase extraction using Oasis HLB cartridges (Waters Ltd. Mississauga ON), filtered, and analyzed by liquid chromatography-electrospray ionization-tandem mass spectrometry (LC/ESI-MS/MS) in positive or negative ionization mode. The analysis of the complete list of analytes (SI Table S1) required a total of five instrumental runs.

Quality assurance steps included analysis of lab blanks for water and plasma along with matrix recovery spikes. Lab blanks for plasma (N = 2) were all less that detection limits except for 2 compounds. DEET was detected at 0.3–1.6 ng/g and venlafaxine was detected in 1 of 2 at 0.16 ng/g. In water blanks (N = 2) DEET (0.87 ng/L) and erythromycin (2.5 ng/L) were both detected in 1 of 2 blank samples. Detections in blanks were close to the detection limits (SI Table S2). Detection limits (DLs) were defined as lowest calibration level adjusted to the maximal sample size or 3 times the signal of the noise in the target channel converted to an equivalent sample, whichever was greater. DLs ranged from 0.08 ng/L to 500 ng/L (median 3 ng/L) for water and 0.016 to 100 ng/g (median 0.6 ng/g) for plasma. Where analytes were detected in two or more samples of the same species or series of water samples, non-detect values were converted to ½ the DL value for reporting purposes. Concentrations for the series of water samples collected at CPM1, CPM2 and JH in January 2014, as well as for individual wild goldfish and carp were averaged using the ½ the DL values. For 6 compounds, ibuprofen, caffeine, diltiazem, diazepam, amitriptyline, and fluoxetine, parent compounds and transformation products (2-hydroxy-ibuprofen, 1,7-dimethylxanthine, desmethyldiltiazem, oxazepam, 10-hydroxy-amitriptyline, norfluoxetine) were summed for graphical presentation but are tabulated individually in Supplementary Information.

Recoveries of unlabeled standards spiked at 15–300 ng/sample in plasma ranged from 56 to 135% for 117 of 127 compounds; unusually high recoveries (150–259% were seen for 4 compounds and low recoveries (15–50%) for 6. In water samples recoveries ranged from 57 to 119% for 119 of 127 compounds; unusually high recoveries (164–222%) were seen for 2 compounds and low recoveries (24–50%) for 6. Further results for DLs, blanks and matrix recoveries are provided in SI Table S2.

### Bioaccumulation calculations

Bioaccumulation factor (BAF_P_) was calculated as concentration in fish plasma (ng/g × 1000) divided by concentration in water (ng/L). Two widely used modeling approaches for predicting organic contaminant bioaccumulation were assessed: the BCF-BAF model for whole body BAFs^53^ and the fish-plasma concentration calculation of Huggett *et al*.^35^ modified by Berninger *et al*.^54^. The latter involves estimating an octanol-water partition coefficient (Kow) adjusted for fraction ionized (log D), using the pKa value to adjust log Kow^54^ taking into account the pH of CPM waters. Further information on the calculation of log D, blood-water partitioning (P_BW_), and plasma concentrations (FPC) is given in the SI.

### Data availability

All data generated or analysed during this study are included in this published article and in Supplementary Information.

## Electronic supplementary material

Supplementary information

## References

[1] Andresen, J. A. *et al*. Emerging pollutants in the north sea in comparison to Lake Ontario, Canada, data. *Environ. Toxicol. Chem.* **26**, 1081–1089 (2007). 10.1897/06-416r.1 17571671

[2] Metcalfe CD, Miao XS, Koenig BG, Struger J (2003). Distribution of acidic and neutral drugs in surface waters near sewage treatment plants in the lower Great Lakes, Canada. Environ. Toxicol. Chem..

[3] Lee, H. B., Peart, T. E., Svoboda, M. L. & Backus, S. Occurrence and fate of rosuvastatin, rosuvastatin lactone, and atorvastatin in Canadian sewage and surface water samples. *Chemosphere* **77**, 1285–1291 (2009). 10.1016/j.chemosphere.2009.09.068 19863993

[4] Lee HB, Peart TE, Svoboda ML (2005). Determination of endocrine-disrupting phenols, acidic pharmaceuticals, and personal-care products in sewage by solid-phase extraction and gas chromatography-mass spectrometry. Journal of Chromatography A.

[5] Li H, Helm PA, Metcalfe CD (2010). Sampling in the great lakes for pharmaceuticals, personal care products, and endocrine-disrupting substances using the passive polar organic chemical integrative sampler. Environ. Toxicol. Chem..

[6] Mayer, T. *et al*. Occurrence of alkylphenolic substances in a Great Lakes coastal marsh, Cootes Paradise, ON, Canada. *Environ Pollut* **147**, 683–690 (2007). 10.1016/j.envpol.2006.09.012 17134807

[7] Mayer T (2008). Dispersal of contaminants from municipal discharges as evidenced from sedimentary records in a great lakes coastal wetland, Cootes Paradise, Ontario. Journal of Great Lakes Research.

[8] Ramirez, A. J. *et al*. Occurrence of pharmaceuticals and personal care products in fish: Results of a national pilot study in the United States. *Environ. Toxicol. Chem.* **28**, 2587–2597 (2009). 10.1897/08-561.1 19320536

[9] Huerta B, Rodríguez-Mozaz S, Barceló D (2012). Pharmaceuticals in biota in the aquatic environment: Analytical methods and environmental implications. Analytical and Bioanalytical Chemistry.

[10] O’Toole S, Metcalfe C (2006). Synthetic musks in fish from urbanized areas of the lower Great Lakes, Canada. Journal of Great Lakes Research.

[11] Uslu, M. O., Biswas, N. & Jasim, S. Chemicals of emerging concern In the Great Lakes region. ID696., 73 pp (International Joint Commission, Windsor, ON, 2012).

[12] Klečka G, Persoon C, Currie R (2010). Chemicals of Emerging Concern in the Great Lakes Basin: An Analysis of Environmental Exposures. Reviews of Environmental Contamination and Toxicology.

[13] Gilroy, È. A. M. *et al*. Halogenated phenolic compounds in wild fish from Canadian Areas of Concern. *Environ*. *Toxicol*. *Chem*. (2017). 10.1002/etc.3781 28256742

[14] Metcalfe, C. D. *et al*. Antidepressants and their metabolites in municipal wastewater, and downstream exposure in an urban watershed. *Environ. Toxicol. Chem.* **29**, 79–89 (2010). 10.1002/etc.27 20821422

[15] Daughton CG, Ternes TA (1999). Pharmaceuticals and personal care products in the environment: Agents of subtle change?. Environmental Health Perspectives.

[16] Du B, Perez-Hurtado P, Brooks BW, Chambliss CK (2012). Evaluation of an isotope dilution liquid chromatography tandem mass spectrometry method for pharmaceuticals in fish. Journal of Chromatography A.

[17] Daughton, C. & Brooks, B. In *In: Environmental contaminants in biota*. *2nd edn*. (eds W. Nelson Beyer & James P. Meador) pp. 286–347 (CRC Press, 2011).

[18] Howard PH, Muir DCG (2011). Identifying New Persistent and Bioaccumulative Organics Among Chemicals in Commerce II: Pharmaceuticals. Environmental Science & Technology.

[19] Chu, S. & Metcalfe, C. D. Analysis of paroxetine, fluoxetine and norfluoxetine in fish tissues using pressurized liquid extraction, mixed mode solid phase extraction cleanup and liquid chromatography-tandem mass spectrometry. *Journal of Chromatography A* **1163**, 112–118 (2007). 10.1016/j.chroma.2007.06.014 17603064

[20] Meador, J. P., Yeh, A., Young, G. & Gallagher, E. P. Contaminants of emerging concern in a large temperate estuary. *Environmental Pollution* **213**, 254–267 (2016). 10.1016/j.envpol.2016.01.088 PMC5509463 26907702

[21] Moreno-González R, Rodríguez-Mozaz S, Huerta B, Barceló D, León VM (2016). Do pharmaceuticals bioaccumulate in marine molluscs and fish from a coastal lagoon?. Environmental Research.

[22] Álvarez-Muñoz, D. *et al*. Occurrence of pharmaceuticals and endocrine disrupting compounds in macroalgaes, bivalves, and fish from coastal areas in Europe. *Environmental Research* **143**, 56–64 (2015). 10.1016/j.envres.2015.09.018 26409498

[23] Xie Z (2015). Occurrence, bioaccumulation, and trophic magnification of pharmaceutically active compounds in Taihu Lake, China. Chemosphere.

[24] Gao, L., Shi, Y., Li, W., Liu, J. & Cai, Y. Occurrence, distribution and bioaccumulation of antibiotics in the Haihe River in China. *Journal of Environmental Monitoring* **14**, 1247–1254 (2012). 10.1039/c2em10916f 22402740

[25] Tanoue R (2015). Uptake and Tissue Distribution of Pharmaceuticals and Personal Care Products in Wild Fish from Treated-Wastewater-Impacted Streams. Environmental Science & Technology.

[26] Scott WC (2016). Predicted and observed therapeutic dose exceedances of ionizable pharmaceuticals in fish plasma from urban coastal systems. Environ. Toxicol. Chem..

[27] Zhao, J.-L. *et al*. Tissue-specific bioaccumulation of human and veterinary antibiotics in bile, plasma, liver and muscle tissues of wild fish from a highly urbanized region. *Environmental Pollution* **198**, 15–24 (2015). 10.1016/j.envpol.2014.12.026 25549863

[28] Du, B. *et al*. Bioaccumulation and trophic dilution of human pharmaceuticals across trophic positions of an effluent-dependent wadeable stream. *Philosophical Transactions of the Royal Society B: Biological Sciences* **369** (2014). 10.1098/rstb.2014.0058 PMC4213599 25313153

[29] Brooks BW (2014). Fish on Prozac (and Zoloft): Ten years later. Aquatic Toxicology.

[30] Brooks, B. W., Huggett, D. B. & Boxall, A. B. A. Pharmaceuticals and personal care products: Research needs for the next decade. *Environ. Toxicol. Chem.* **28**, 2469–2472 (2009). 10.1897/09-325.1 19908931

[31] Caldwell DJ, Mastrocco F, Margiotta-Casaluci L, Brooks BW (2014). An integrated approach for prioritizing pharmaceuticals found in the environment for risk assessment, monitoring and advanced research. Chemosphere.

[32] Simmons, D. *et al*. Reduced anxiety is associated with the accumulation of six serotonin reuptake inhibitors in wastewater treatment effluent exposed, goldfish Carassius auratus. *Scientific Reports* doi:10.1038/s41598-017-15989-z (2017). 10.1038/s41598-017-15989-z PMC5717243 29208964

[33] Simmons, D. *et al*. Altered expression of metabolites and proteins in wild and caged fish exposed to wastewater effluents *in situ*. *Scientific Reports*, doi:10.1038/s41598-017-12473-6 (2017). 10.1038/s41598-017-12473-6 PMC5717254 29208926

[34] Kelton, N., Chow-Fraser, P. & Jordan, I. Relationship between sediment phosphorus release rates and characteristics of the benthic microbial community in a hypereutrophic marsh. *Aquatic Ecosystem Health & Management* **7**, 31–41 (2004).

[35] Huggett DB, Cook JC, Ericson JF, Williams RT (2003). A Theoretical Model for Utilizing Mammalian Pharmacology and Safety Data to Prioritize Potential Impacts of Human Pharmaceuticals to Fish. Human and Ecological Risk Assessment: An International Journal.

[36] Brown, J. N., Paxéus, N., Förlin, L. & Larsson, D. G. J. Variations in bioconcentration of human pharmaceuticals from sewage effluents into fish blood plasma. *Environmental Toxicology and Pharmacology* **24**, 267–274 (2007). 10.1016/j.etap.2007.06.005 21783821

[37] Fick J (2010). Therapeutic levels of levonorgestrel detected in blood plasma of fish: results from screening rainbow trout exposed to treated sewage effluents. Environmental Science and Technology.

[38] US EPA. Method 1694: Pharmaceuticals and Personal Care Products in Water, Soil, Sediment, and Biosolids by HPLC/MS/MS. EPA-821-R-08-002. (US Environmental Protection Agency, Office of Science and Technology, Engineering and Analysis Division (4303T), Washington, DC, 2007).

[39] Chu S, Metcalfe CD (2007). Simultaneous determination of triclocarban and triclosan in municipal biosolids by liquid chromatography tandem mass spectrometry. Journal of Chromatography A.

[40] de Solla SR (2016). Bioaccumulation of pharmaceuticals and personal care products in the unionid mussel Lasmigona costata in a river receiving wastewater effluent. Chemosphere.

[41] Niagara Region. *Grimsby Water Treatment Plant System* http://www.regional.niagara.on.ca/2041/master-servicing-plan/default.aspx., 2016).

[42] Lindholm-Lehto, P. C., Ahkola, H. S. J., Knuutinen, J. S. & Herve, S. H. Widespread occurrence and seasonal variation of pharmaceuticals in surface waters and municipal wastewater treatment plants in central Finland. *Environmental Science and Pollution Research* **23**, 7985–7997 (2016). 10.1007/s11356-015-5997-y 26769590

[43] aus der Beek T (2016). Pharmaceuticals in the environment—Global occurrences and perspectives. Environmental Toxicology and Chemistry.

[44] Du, B. *et al*. Bioaccumulation of human pharmaceuticals in fish across habitats of a tidally influenced urban bayou. *Environ. Toxicol. Chem.* **35**, 966–974 (2016). 10.1002/etc.3221 26587912

[45] Overturf CL, Overturf MD, Huggett DB (2016). Bioconcentration and endocrine disruption effects of diazepam in channel catfish, Ictalurus punctatus. Comp Biochem Physiol C Toxicol Pharmacol.

[46] Zhao J-L (2017). Uptake and Disposition of Select Pharmaceuticals by Bluegill Exposed at Constant Concentrations in a Flow-Through Aquatic Exposure System. Environmental Science & Technology.

[47] Nallani, G. C., Paulos, P. M., Constantine, L. A., Venables, B. J. & Huggett, D. B. Bioconcentration of ibuprofen in fathead minnow (Pimephales promelas) and channel catfish (Ictalurus punctatus). *Chemosphere* **84**, 1371–1377 (2011). 10.1016/j.chemosphere.2011.05.008 21658739

[48] Hughes SR, Kay P, Brown LE (2013). Global synthesis and critical evaluation of pharmaceutical data sets collected from river systems. Environmental Science and Technology.

[49] Liu J (2015). Occurrence, bioaccumulation and risk assessment of lipophilic pharmaceutically active compounds in the downstream rivers of sewage treatment plants. Science of The Total Environment.

[50] Smith EM (2012). *In vitro* inhibition of cytochrome P450-mediated reactions by gemfibrozil, erythromycin, ciprofloxacin and fluoxetine in fish liver microsomes. Aquatic Toxicology.

[51] Lyssimachou A, Thibaut R, Gisbert E, Porte C (2014). Gemfibrozil modulates cytochrome P450 and peroxisome proliferation-inducible enzymes in the liver of the yellow European eel (Anguilla anguilla). Environmental Science and Pollution Research.

[52] Donnachie, R. L., Johnson, A. C. & Sumpter, J. P. A rational approach to selecting and ranking some pharmaceuticals of concern for the aquatic environment and their relative importance compared with other chemicals. *Environ. Toxicol. Chem.* (2015). 10.1002/etc.3165 26184376

[53] US EPA. Exposure Assessment Tools and Models, Estimation Program Interface (EPI) Suite Ver 4.1. (US Environmental Protection Agency, Office of Pollution Prevention and Toxics, Washington, DC, 2011).

[54] Berninger JP (2011). Effects of the antihistamine diphenhydramine on selected aquatic organisms. Environ. Toxicol. Chem..

[55] Arnot, J. A. *et al*. A quantitative structure-activity relationship for predicting metabolic biotransformation rates for organic chemicals in fish. *Environ. Toxicol. Chem.* **28**, 1168–1177 (2009). 10.1897/08-289.1 19152232

[56] Schulz M, Iwersen-Bergmann S, Andresen H, Schmoldt A (2012). Therapeutic and toxic blood concentrations of nearly 1,000 drugs and other xenobiotics. Critical Care.

[57] Nichols JW (2015). Observed and modeled effects of pH on bioconcentration of diphenhydramine, a weakly basic pharmaceutical, in fathead minnows. Environ. Toxicol. Chem..

[58] Environment Canada. Major Ions and Nutrients, vol. 1. (National Laboratory for Environmental Testing, Burlington, ON, Canada, 1994).

[59] AXYS. Method MLA-075: Analysis of pharmaceutical and personal care products and hormones in solid, aqueous, tissue and POCISs samples by LC-MS/MS. (AXYS Analytical Services Ltd, Sidney, BC, 2015).

[60] Berninger JP, Brooks BW (2010). Leveraging mammalian pharmaceutical toxicology and pharmacology data to predict chronic fish responses to pharmaceuticals. Toxicology Letters.

