# Supplementary material for: Bioaccumulation of pharmaceuticals and personal care product chemicals in fish exposed to wastewater effluent in an urban wetland
Source: Sci Rep. 2017 Dec 5;7:16999. doi: 10.1038/s41598-017-15462-x (PMC5717258; doi:10.1038/s41598-017-15462-x)
Supplement: Supplementary file 1 — Supplementary information [file 41598_2017_15462_MOESM1_ESM.pdf]

Supplementary information

Bioaccumulation of pharmaceuticals and personal care product chemicals in fish exposed to wastewater effluent in an urban wetland

Derek Muir, Denina Simmons, Xiaowa Wang, Tom Peart, Maria Villella, Jason Miller and Jim Sherry

Aquatic Contaminants Research Division, Water and Science Technology Directorate, Environment & Climate Change Canada, Burlington, Ontario

Corresponding author:

Derek Muir
Aquatic Contaminants Research Division,
Environment & Climate Change Canada,
867 Lakeshore Road, Burlington ON L7S 1A1 Canada
Ph: 905-319-6921

Additional quality assurance information:

Effect of storage times on measured surface water concentrations:

Samples collected in January 2014 were stored at $4^{o}C$ in the dark and analysed within 6 weeks of collection while those from August 2012 were held at $-20^{o}C$. Samples collected in July 2014 were analysed in February 2015 after 6 months storage at $4^{o}C$ in the dark. PPCP profiles and concentrations were generally very similar in both January, August, and July samples for CPM3. This is illustrated by the ratios of concentrations for January/July 2014 and for August 2012/July 2014 samples. Detection frequency was identical in all 3 sampling times (Table S1). Median ratios were 2.1 and 1.5 for January/July 2014 and for August 2012/July 2014, respectively. For January-July, Naproxen, diphenhydramine, clarithromycin, benzoylecgonine (cocaine), and venlafaxine had ratios ranging from 19 to 54 while all others ranged from 0.3 to 10. For August 2012/July 2014 the ratios ranged from 0.3 to 10. While these differences could be due in part to storage conditions, because maximum storage times of 40 days recommended by the USEPA Method 1694[1] , were exceeded for the July2014 samples, they could also be due to the seasonal variation in PPCP use and greater rates of degradation in summer. Lindholm-Lehto et al.[2] and Golovko et al.[3] noted higher PPCP concentrations in waste waters and receiving waters in winter. Furlong et al[4] found median recoveries of approximately 65% for 110 pharmaceuticals in surface water samples following 30 days storage at 4 ^{o}C. There appear to be no very long term (>30 days) studies of storage stability of PPCPs in surface waters at 4 ^{o}C.

Plasma sample extractions:

Plasma was separated from the blood samples by centrifugation (9300g, 4 min) at 4 °C, transferred to cryogenic vials, flash frozen in liquid nitrogen, and subsequently stored at −80 °C prior to analysis. Further details are given in Simmons et al. [5,6]

Additional information on Bioaccumulation calculations:

1. Log D calculations

$\log D_{bases} = \log Kow + \log [1 /(1+10^{(pKa-pH)}]$; $\log D_{acids} = \log P + \log [1/(1+10^{(pH-pKa)})$

2. Blood-water partitioning (P_{BW}):[7,8]

$\text{Log PBW} = \log ((10^{0.73*\log D*0.16}) + 0.84)$

3. Fish plasma concentrations (FPC)

$FPC = P_{BW} * \text{water concentration (ng/mL)}$

Table S1. Results for PPCPs in water (ng/L) for samples collected in Cootes Paradise Marsh (CPM1 - 3) and in Jordan Harbour (JH) reference site and ratio of concentrations at CPM3 for January and July 2014

Location and sampling date ->	CPM1 Jan 21, 2014	CPM1 Jan 24 2014	CPM1 Jan 29, 2014	CPM1 Feb 3, 2014	CPM1 Jul 16, 2014	CPM2 Aug 13, 2012	CPM3 Aug 13, 2012	CPM3 Jan 21, 2014	CPM3 Jul 16, 2014	CPM3 Jan-Jul ratio[1]	CPM3 Aug-Jul ratio	JH Jan 21, 2014	JH Jan 29, 2014	JH Jul 16, 2014
Sample Size (L) ->	0.942	0.949	0.862	0.978	0.965	0.948	0.986	0.964	0.965			0.936	0.960	0.980
UNITS ->	ng/L	ng/L	ng/L	ng/L	ng/L	ng/L	ng/L	ng/L	ng/L			ng/L	ng/L	ng/L
1,7-Dimethylxanthine	2000	124	3630	951	162	247	44.3	63	31.1	2.0	1.4	<64.1	<62.5	<61.2
10-hydroxy-amitriptyline	26.9	0.359	32.6	1.48	0.216	1.37	0.056	0.184	0.078	2.4	0.7	<0.0799	<0.0692	<0.153
2-Hydroxy-ibuprofen	8370	42.15	12100		41.45	917	40.6	196	41.5	4.7	1.0	<85.5	<83.4	<81.6
4-Epianhydrochlor-tetracycline	<63.7	<63.3	<69.6	<61.4	<62.2	<61	<60.8	<62.2	<62.2	-	-	<64.1	<62.5	<61.2
4-Epianhydrotetra-cycline	<15.9	<15.8	<17.4	<15.3	<15.5	<15.2	<15.2	<15.6	<15.5	-	-	<16	<15.6	<15.3
4-Epichlortetracycline	<15.9	<15.8	<17.4	<15.3	<15.5	<15.2	<15.2	<15.6	<15.5	-	-	<16	<15.6	<15.3
4-Epioxytetracycline	<6.37	<6.33	<6.96	<6.14	<6.22	<6.1	<6.08	<6.22	<6.22	-	-	<6.7	<6.25	<7.47
4-Epitetracycline	<6.37	<6.33	<6.96	<6.14	<6.22	<6.1	<6.08	<7.27	<6.22	-	-	<6.41	<6.25	<8
Acetaminophen	<15.9	<15.8	<17.4	<17.3	<15.5	<15.8	<15.2	<15.6	<15.5	-	-	<16	<15.6	<15.3
Alprazolam	0.217	0.158	0.153	0.349	0.156	<0.191	<0.26	<0.311	<0.311	-	-	<0.107	<0.104	<0.306
Amitriptyline	35.7	54.8	42	54.3	2.33	6.63	0.848	0.948	0.156	6.1	5.4	<0.127	<0.186	<0.306
Amlodipine	6.43	6.18	14.2	18.4	0.775	<0.527	<0.506	<1.56	<1.55	-	-	<0.534	<0.521	<1.53
Amsacrine	<0.235	<0.514	<0.383	<0.54	<0.327	<0.232	<0.139	<0.546	<0.409	-	-	<0.254	<0.108	<0.464
Anhydrochlortetra-cycline	<15.9	<15.8	<17.4	<15.3	<15.5	<15.2	<15.2	<15.6	<15.5	-	-	<16	<15.6	<15.3
Anhydrotetracycline	<15.9	<15.8	<17.4	<15.3	<15.5	<15.2	<15.2	<15.6	<15.5	-	-	<16	<15.6	<15.3
Azathioprine	<5.69	<12.1	<4.39	<3.68	<2.07	<2.92	<2.03	<2.07	<2.07	-	-	<3.15	<2.39	<2.04
Azithromycin	1070	418	1090	536	44.3	0.945	0.76	7.57	0.775	9.8	1.0	<1.93	<1.56	<1.69
Benzoylecgonine	305	16.2	402	460	6.69	7.92	0.300	8.83	0.393	22.5	0.8	1.12	1.09	0.153
Benztropine	0.493	0.264	0.58	0.256	0.583	0.4	0.401	0.259	0.259	1.0	1.5	0.339	0.386	0.255
Betamethasone	<31.5	<1.58	<16.7	<1.53	<1.55	<11.2	<9.96	<5.18	<5.18	-	-	<0.534	<6.23	<5.1
Bisphenol A	2360	264	1940	256	259	<527	<507	<518	<518	-	-	<534	<521	<510
Busulfan	<4.25	<4.97	<4.64	<7.51	<5.34	<4.22	<4.06	<4.15	<4.15	-	-	<4.27	<4.17	<4.08

Caffeine	1060	40.5	3280	1110	71	59.2	54.7	33.6	25.7	1.3	2.1	38.7	18.7	17
Carbadox	<1.59	<3.68	<11.2	<5.4	<2.61	<1.58	<1.52	<2.95	<4.0	-	-	<1.6	<1.56	<3.01
Carbamazepine	203	139	248	174	184	299	52.9	4.83	8.46	0.6	6.3	2.63	2.27	0.765
Cefotaxime	<252	<6.33	<262	<6.14	<6.22	<182	<126	<6.22	<6.22	-	-	<6.41	<90.2	<6.12
Chlortetracycline [CTC]	<21.2	<6.67	<23.2	<6.89	<6.8	<20.3	<20.3	<7.26	<7.09	-	-	<21.4	<20.8	<7.21
Ciprofloxacin	93	79.5	77.5	105	8.25	9.05	7.3	7.10	9.50	0.7	0.8	<176	<9.1	<17.2
Citalopram	212	260	232	296	37.9	28.5	2.24	5.94	0.785	7.6	2.9	1.38	0.835	0.204
Clarithromycin	546	930	887	1940	136	45.3	2.87	26.1	0.775	33.7	3.7	<1.6	<1.56	<1.53
Clinafloxacin	25.75	19.3	31.75	22.8	17.2	22.1	24.05	18.9	16.3	1.2	1.5		<39.6	<33.2
Clotrimazole	1.72	1.64	2.34	1.33	0.46	0.211	0.203	0.208	0.208	1.0	1.0	<0.427	<0.417	<0.408
Cloxacillin	<25.8	<3.16	45.6	<6.53	<3.11	<5.7	<4.5	<3.11	<3.11	-	-	<3.26	<3.13	<3.06
Cocaine	65.7	0.079	100	51.5	0.0775	<0.0527	<0.197	<0.156	<0.155	-	-	<0.221	<0.158	<0.174
Colchicine	<0.849	<2.95	<0.928	<2.25	<3.32	<1.39	<2.82	<3.09	<0.829	-	-	<0.855	<0.834	<0.816
Cyclophosphamide	0.735	1.61	0.915	0.409	6.15	<0.844	<0.843	<0.829	<0.829	-	-	<0.855	<0.834	<0.816
Daunorubicin	<9.58	<8.43	<9.28	<8.18	<8.29	<8.67	<8.34	<8.29	<8.29	-	-	<10.8	<8.34	<8.16
DEET	230	57.8	470	241	93.4	92.1	52.1	27.8	81.9	0.3	0.6	13.5	25.3	24.9
Dehydronifedipine	15.8	9.28	18.4	12	24.7	18.9	2.68	0.634	1.03	0.6	2.6	<0.642	<0.625	<0.678
Demeclocycline	<15.9	<15.8	<17.4	<15.3	<15.5	<15.2	<15.2	<15.6	<15.5	-	-	<16	<15.6	<15.3
Desmethyldiltiazem	318	65.8	312	99	0.078	0.126	<0.0691	<0.156	<0.155	-	-	<0.0738	0.0773	<0.153
Diatrizoic acid	103	93.4	239	421	320	179	<58.8	<24.9	<24.9	-	-	<25.6	<38.6	<24.5
Diazepam	0.375	0.854	0.957	1.06	0.795	1.45	<0.485	<0.311	<0.373	-	-	<0.107	<0.104	<0.306
Digoxigenin	<204	<38.2	<178	<47.3	<47.7	<68.8	<65.9	<27.9	<25.1	-	-	<57.8	<43.6	<24.3
Digoxin	<6.37	<6.33	<6.96	<6.14	<6.22	<6.33	<6.08	<6.22	<6.22	-	-	<6.41	<6.25	<6.12
Diltiazem	765	322	911	583	1.28	0.525	0.645	0.621	0.210	3.0	3.1	<0.809	<1.13	<0.398
Diphenhydramine	400	58.6	427	345	31.5	39.7	1.30	7.62	0.311	24.5	4.2	<0.641	<0.625	<0.612
Doxorubicin	<25.5	<25.3	<27.8	<24.6	<24.9	<25.3	<24.3	<24.9	<24.9	-	-	<25.6	<25	<24.5
Doxycycline	<8.9	<6.33	<9.95	<6.24	<6.22	<6.16	<6.61	<6.38	<6.22	-	-	<6.51	<6.25	<6.56
Drospirenone	<8.49	<8.43	10	<8.18	<8.29	<8.56	<8.65	<8.29	<8.29	-	-	<8.55	<8.34	<8.16
Enrofloxacin	<3.72	<3.16	<4.02	<3.07	<3.11	<3.16	<3.45	<3.11	<3.11	-	-		<3.56	<3.06
Erythromycin-H2O	43.1	42.5	44.5	89.7	6.21	62.2	6.56	3.86	2.46	1.6	2.7	4.03	3.49	2.66
Etoposide	NQ	<2.11	NQ	<2.05	<2.07	<2.62	<2.03	<2.07	<2.07	-	-	<2.14	<2.08	<2.04

Flumequine	<11.3	<3.45	<15.4	<4.29	<4.73	<7.77	<4.38	<2.39	<2.48	-	-	<2.61	<4.2	<1.71
Fluocinonide	<2.12	<6.33	<2.32	<6.14	<6.22	<2.11	<2.03	<6.22	<6.22	-	-	<2.13	<2.08	<6.12
Fluoxetine	19.8	19.5	25.1	20.9	4.49	0.79	0.76	0.78	0.775	1.0	1.0	<1.6	<1.56	<1.53
Fluticasone propionate	1.80	2.27	3.46	3.91	1.04	<1.43	<0.771	<2.09	<2.39	-	-	<0.711	<0.694	<2.43
Furosemide	507	349	694	601	20.7	<42.2	<40.6	<41.5	<41.5	-	-	<42.7	<41.7	<40.8
Gemfibrozil	41.3	4.75	26.4	20.7	0.775	5.49	0.760	0.780	0.775	1.0	1.0	0.8	1.6	0.765
Glipizide	<6.37	<6.33	<6.96	<6.14	<6.22	<6.33	<6.08	<6.22	<6.22	-	-	<6.41	<6.25	<6.12
Glyburide	<3.18	<3.16	<3.48	<3.07	<3.11	<3.16	<3.04	<3.11	<3.11	-	-	<3.2	<3.13	<3.06
Hydrochlorothiazide	128	91.3	166	198	30.6	<12.7	<12.2	<20.7	<20.7	-	-	<12.8	<12.5	<20.4
Hydrocortisone	<61.5	<63.3	<58.8	<61.4	<62.2	<42.7	<27	<62.2	<62.2	-	-	<31.4	<20.8	<61.2
Ibuprofen	3420	7.90	3990	NQ	7.75	59.1	<15.2	<15.6	<15.5	-	-	<16	<15.6	<15.3
Iopamidol	68.5	220	122	783	280	349	211	768	643	1.2	0.3	85.5	47.05	452
Isochlortetracycline	<6.37	<6.33	<6.96	<6.14	<6.22	<6.1	<6.08	<6.22	<6.22	-	-	<6.41	<6.25	<6.12
Lincomycin	3.93	1.58	14.7	10.1	1.56	<3.16	<3.04	<3.11	<3.11	-	-	17.1	6.22	1.53
Lomefloxacin	<3.38	<5.15	<8.13	<4.97	<5.58	<4.22	<6.4	<3.11	<4.7	-	-		<4.38	<3.75
Medroxyprogesterone Acetate	4.86	2.11	6.43	2.05	2.07	<4.22	<4.06	<4.15	<4.15	-	-	<4.27	<4.17	<4.08
Melphalan	233	29.0	210	24.4	20.6	<235	<133	<35.1	<24.9	-	-	<122	<148	<24.5
Meprobamate	5.66	8.53	4.39	7.86	10.6	17.6	6.14	2.08	2.08	1.0	3.0	2.94	4.19	2.04
Methylprednisolone	<15.1	<10.3	<25.9	<16	<12.2	<3.77	<12.7	<8.62	<4.15	-	-	<15.1	<3.48	<4.08
Metoprolol	292	301	358	423	184	161	2.94	8.55	2.57	3.3	1.1	<6.02	<4.17	<4.25
Metronidazole	64.5	4.62	103	103	7.4	<4.88	<4.06	<6.17	<8.1	-	-	<4.33	<4.3	<6.56
Miconazole	2.73	0.79	3.48	0.865	0.775	<1.58	<1.52	<1.56	<1.55	-	-	<1.6	<1.56	<1.53
Minocycline	<63.7	<69.8	<69.6	<70	<69.2	<61	<60.8	<69.8	<67.5	-	-	<64.1	<62.5	<70.1
Moxifloxacin	<33.7	<17.6	<32.4	17.1	<8.84	<27.2	<14.3	<4.65	<4.48	-	-	<42.8	<10.5	<6.23
Naproxen	3230	1.58	4300	300	1.56	17	5.11	85	1.56	54.5	3.3	6.27	1.565	1.53
Norfloxacin	<74.4	<53.8	<53.6	<46	<59.9	<72.6	<37.1	<18.8	<58	-	-		<31.7	<30.8
Norfluoxetine	4.17	2.56	6.27	5.09	<1.55	<0.527	<0.506	<1.56	<1.55	-	-	<0.534	<0.521	<1.53
Norgestimate	<35.8	<7.48	<18.8	<10.9	<7.79	<9.05	<9.03	<5.61	<6.03	-	-	<7.81	<4.77	<4.38
Norverapamil	1.09	0.769	0.765	1.3	0.483	<0.0527	<0.0612	<0.156	<0.155	-	-	<0.0534	<0.0523	<0.153
Ofloxacin	109	133	112	128	4.2	<1.62	<1.52	<1.56	<1.55	-	-		<1.56	<1.53

Ormetoprim	<0.637	<0.633	<0.696	<0.614	<0.622	<0.633	<0.608	<0.622	<0.622	-	-	<0.641	<0.625	<0.612
Oxacillin	<5.92	<3.16	<7.75	<3.07	<3.11	<3.16	<3.04	<3.11	<3.11	-	-	<3.2	<3.13	<3.06
Oxazepam	135	105	162	151	43.3	78.2	10.8	4.39	2.08	2.1	5.2	<4.27	<4.17	<4.08
Oxolinic Acid	<0.637	<0.633	<2.3	<0.614	<0.622	<2.39	<1.13	<0.622	<0.622	-	-	<1.26	<0.96	<0.612
Oxytetracycline [OTC]	<21.2	<6.33	<23.2	<6.14	<6.22	<20.3	<20.3	<6.31	<6.22	-	-	<21.4	<20.8	<6.95
Paroxetine	6.61	9.17	10.4	9.51	2.07	<1.41	<1.53	<4.15	<4.15	-	-	<1.42	<1.39	<4.08
Penicillin G	46	1.58	50.3	1.54	1.56	<7.64	<5.85	<3.11	<3.11	-	-	<3.2	<3.13	<3.06
Penicillin V	1.59	1.77	2.81	8.77	1.57	<7.77	<6.04	<3.11	<3.11	-	-	<3.67	<3.13	<3.06
Prednisolone	<47.8	<38.1	<85.1	<36.9	<30.3	<20.4	<21.3	<50.6	<45.4	-	-	<23.2	<2.08	<6.12
Prednisone	<175	<43.2	<166	<89.1	<74.5	<97.8	<115	<29.4	<56	-	-	<45	<30.9	<35.5
Promethazine	<2.07	<1.4	<1.27	<1.36	<1.38	<0.372	<0.281	<1.38	<1.38	-	-	<0.331	<0.152	<1.36
Propoxyphene	<0.106	<0.316	<0.209	<0.307	<0.311	0.186	<0.101	<0.311	<0.311	-	-	<0.107	<0.104	<0.306
Propranolol	40.8	45.8	55.7	64.7	1.04	4.38	<0.675	<2.07	<2.07	-	-	<0.711	<0.694	<2.04
Rosuvastatin	113	8.86	147	263	2.07	<4.22	<4.06	<4.15	<4.15	-	-	<4.27	<4.17	<4.08
Roxithromycin	<0.322	<1.43	<0.347	<0.603	0.5	<0.316	<0.304	<0.311	<0.311	-	-	<0.32	<0.313	<0.306
Sarafloxacin	<15.9	<15.8	<17.4	<15.3	<15.5	<15.8	<15.2	<15.6	<15.5	-	-		<15.6	<15.3
Sertraline	29	28.1	40.3	40.3	2.12	1.45	0.358	1.26	1.22	1.0	0.3	0.343	0.163	1.1
Simvastatin	<7.07	<21.1	<7.72	<20.5	<20.7	<7.03	<6.75	<20.7	<20.7	-	-	<7.11	<6.94	<20.4
Sulfachloropyridazine	NQ[2]	<1.58	NQ	<1.53	<1.55	<6.38	<1.52	<1.56	<1.55	-	-	<1.6	<1.56	<1.53
Sulfadiazine	NQ	<1.58	NQ	<1.69	<1.55	<1.58	<1.52	<1.56	<1.55	-	-	<1.6	<1.56	<1.53
Sulfadimethoxine	NQ	4.57	NQ	9.55	0.1575	0.379	0.275	0.459	0.182	2.5	1.5	<0.908	<0.313	<0.454
Sulfamerazine	NQ	<1.45	NQ	<2.06	<1.15	<0.633	<0.837	<0.849	<0.901	-	-	<1.21	<0.625	<0.715
Sulfamethazine	<0.637	<3.34	<257	<5.65	<1.79	<3.86	0.304	<2.08	<2.01	-	-	<2.96	<1.92	<2.02
Sulfamethizole	NQ	<0.967	NQ	<1.58	<0.859	<2.07	<0.608	<0.787	<0.719	-	-	<1.64	<0.625	<0.787
Sulfamethoxazole	290	441	NQ	311	21.3	31.7	3.99	19.8	2.56	7.7	1.6	3	4.08	0.57
Sulfanilamide	NQ	<158	NQ	<181	<155	<15.8	<15.2	<156	<155	-	-	<16	<15.6	<153
Sulfathiazole	NQ	<1.58	NQ	<1.53	<1.78	<1.58	<1.52	<1.56	<1.55	-	-	<1.6	<1.56	<1.53
Tamoxifen	<0.425	<0.422	<0.464	<0.409	<0.414	<0.422	<0.406	<0.415	<0.415	-	-	<0.427	<0.417	<0.408
Teniposide	NQ	<4.22	NQ	<4.09	<4.14	<4.22	<4.06	<4.15	<4.15	-	-	<4.27	<4.17	<4.08
Tetracycline [TC]	<21.2	<6.33	<23.2	<6.14	<6.22	<20.3	<20.3	<6.22	<6.22	-	-	<21.4	<20.8	<6.12
Theophylline	2060	311	2410	1340	229	<291	<75.3	89.7	<62.2	-	-	<74.8	<71.9	<61.2

Thiabendazole	13.8	14.6	16.2	14.2	9.11	9.05	<1.52	<1.56	<1.55	-	-	<1.6	<1.56	<1.53
Trenbolone	<4.57	<4.22	<6.29	<4.09	<4.14	<1.41	<1.35	<4.15	<4.15	-	-	<2.72	<1.39	<4.08
Trenbolone acetate	<0.665	<0.316	<0.55	<0.337	<0.339	<0.492	<0.417	<0.311	<0.324	-	-	<0.107	<0.104	<0.306
Triclocarban	10.3	7.75	12.1	9.07	1.56	<3.16	<3.04	<3.11	<3.11	-	-	<3.2	<3.13	<3.06
Triclosan	160	31.7	256	147	31.1	<63.3	<60.8	<62.2	<62.2	-	-	<64.1	<62.5	<61.2
Trimethoprim	186	87.9	246	235	2.79	12.9	<3.48	<1.56	<1.55	-	-	<2.24	<1.56	<1.53
Tylosin	<6.37	<6.33	<6.96	<6.14	<6.22	<6.33	<6.08	<6.22	<6.22	-	-	<6.41	<6.25	<6.12
Valsartan	3660	12.3	3050	751	4.29	465	3.26	5.63	2.08	2.7	1.6	6.33	2.87	2.04
Venlafaxine	413	522	489	573	57	119	8.28	14.9	0.796	18.7	10.4	1.91	1.56	0.204
Verapamil	5.36	5.57	6.31	7.45	0.243	<0.111	<0.051	<0.156	0.078	-	-	<0.065	<0.060	<0.153
Virginiamycin M1	<40.1	<3.16	<40.2	<5.61	<3.58	<17.3	<11.4	<3.11	<3.11	-	-	<11.7	<4.72	<3.06
Warfarin	2.98	13.5	4.34	5.21	0.775	<1.58	<1.52	<1.56	<1.55	-	-	<1.6	<1.56	<1.53
Zidovudine	48	12.7	79.2	59.1	12.5	<25.3	<24.3	<24.9	<24.9	-	-	<25.6	<25	<24.5
ΣAmitriptyline[3]	62.6	74.6	55.2	55.8	2.5	8.0	0.9	1.1	0.2	5.5	4.5	<0.1	<0.1	<0.1
ΣIbuprofen[3]	11790	16090	50.1	0.0	49.2	976	40.6	196	41.5	4.7	1.0	<15	<15	<15
ΣCaffeine[3]	3060	6910	165	2061	233	306	99.0	96.6	56.8	1.7	1.7	38.7	18.7	17.0
ΣDiltiazem[3]	1083	1223	388	682	1.4	0.7	0.6	0.6	0.2	3.0	3.0	<0.1	0.08	<0.1
ΣDiazepam[3]	135	163	106	152	44.1	79.7	10.8	4.4	2.1	2.1	5.1	<0.1	<0.1	<0.1
ΣFluoxetine[3]	24.0	31.4	22.1	26.0	4.5	0.8	0.8	0.8	0.8	1.0	1.0	<0.5	<0.5	<0.5
Σcocaine[3]	371	502	16.3	512	6.8	7.9	0.3	8.8	0.4	22.0	0.8	1.12	1.09	0.15
Number detected	63	64	64	63	64	39	32	32	32			16	17	16

[1]Ratio of concentrations at CPM3 for January and July 2014 and for August 2012 and July 2014 samples. Dashes indicate non-detectable values prevented ratio calculation

[2]NQ = not quantified

[3]ΣAmitriptyline = sum of amitriptyline and10-hydroxy-amitriptyline; ΣIbuprofen = sum of ibuprofen and 2-hydroxy-ibuprofen; ΣCaffeine = sum of caffeine + 1,7-dimethylxanthine; ΣDiltiazem = sum of diltiazem and desmethyldiltiazem; ΣDiazepam = sum of diazepam and oxazepam; ΣFluoxetine = sum of fluoxetine and norfluoxetine; Σcocaine = sum of cocaine and benzoylecgonine

Table S2. Detection limits, lab blanks and matrix recoveries for 127 PPCPs in water and plasma[1]

	Water	Water	Water	Plasma	Plasma	Plasma
	DL range[2]	Blank range	Average recovery	DL range[2]	Blank range	Average recovery
	ng/L	ng/L	%	ng/g	ng/g	% ± SD
Analyte			N=2			N=4
Acetaminophen	<15	<15	98	<3-<6	<3-<6	98 ± 4
Alprazolam	<0.3	<0.3	93	<0.06-<0.12	<0.06-<0.12	88 ± 30
Amitriptyline	<0.3	<0.3	97	<0.06-<0.12	<0.06-<0.12	99 ± 4
Amlodipine	<1.5	<1.5	91	<0.3-<0.6	<0.3-<0.6	114 ± 15
Amsacrine	<0.08	<0.08	96	<0.016-<0.031	<0.016-<0.031	108 ± 19
Anhydrochlortetracycline [ACTC]	<15	<15	74	<3-<6	<3-<6	37 ± 16
Anhydrotetracycline [ATC]	<15	<15	75	<3-<6	<3-<6	77 ± 18
Azathioprine	<2	<2	98	<0.4-<0.74	<0.4-<0.74	95 ± 10
Azithromycin	<1.5	<1.5	68	<0.3-<0.6	<0.3-<0.6	149 ± 36
Benzoylecgonine	<0.3	<0.3	111	<0.06-<0.12	<0.06-<0.12	99 ± 3
Benztropine	<0.5-<0.7	<0.5-<0.7	96	<0.1-<0.28	<0.1-<0.28	100 ± 2
Betamethasone	<5-<1.5	<5-<1.5	108	<0.42-<0.60	<0.42-<0.6	124 ± 36
Bisphenol A	<500	<500	107	<100-<200	<100-<200	95 ± 5
Busulfan	<4	<4	107	<0.8-<1.33	<0.8-<1.33	98 ± 11
Caffeine	<15	<15	79	<3-<6	<3-<6	100 ± 34
Carbadox	<1.5	<1.5	72	<0.3-<0.6	<0.3-<0.6	49 ± 19
Carbamazepine	<1.5	<1.5	104	<0.3-<0.6	<0.3-<0.6	101 ± 15
Cefotaxime	<6-<22.5	<6-<22.5	108	<8.59	NQ -<8.59	205 ± 110
Chlortetracycline [CTC]	<6-<20	<6-<20	49	<1.2-<8.89	<1.2-<8.89	108 ± 37
Ciprofloxacin	<6	<6	99	<1.2-<2.4	<1.2-<2.4	106 ± 19
Citalopram	<0.4-<0.8	<0.4-<0.8	92	<0.08-<0.13	<0.08-<0.13	96 ± 16
Clarithromycin	<1.5	<1.5	94	<0.3-<0.6	<0.3-<0.6	116 ± 18
Clinafloxacin	<6-<24.4	<6-<24.4	111	<1.2-<8.68	<1.2-<8.68	112 ± 35
Clotrimazole	<0.4	<0.4	92	<0.08-<0.13	<0.08-<0.13	100 ± 3
Cloxacillin	<3	<3	93	< 0.6-<1.2	< 0.6-<1.2	107 ± 31
Cocaine	<0.15	<0.15	104	<0.03-<0.06	<0.03-<0.06	97 ± 4
Colchicine	<0.8	<0.8	101	<0.16-<0.27	<0.16-<0.27	101 ± 10
Cyclophosphamide	<0.8	<0.8	94	<0.16-<0.27	<0.16-<0.27	96 ± 5
Daunorubicin	<8	<8	89	<1.6-<2.67	<1.6-<2.67	80 ± 14
DEET	<0.8-0.87	<0.8-0.87	106	0.367-1.64	0.367-1.64	111 ± 21
Dehydronifedipine	<0.6	<0.6	102	<0.12-<0.24	<0.12-<0.24	103 ± 3
Demeclocycline	<15	<15	82	<3-<6	<3-<6	71 ± 20
Desmethyldiltiazem	<0.15	<0.15	37	<0.03-<0.06	<0.03-<0.06	116 ± 8
Diatrizoic acid	<24	<24	92	<4.8-<8	<4.8-<8	105 ± 6
Diazepam	<0.3	<0.3	101	<0.06-<0.12	<0.06-<0.12	108 ± 6
Digoxigenin	<6	<6	82	<1.2-<2.4	<1.2-<2.4	96 ± 4

Digoxin	<6	<6	76	<1.2-<2.4	<1.2-<2.4	115 ± 10
Diltiazem	<0.3-<1.05	<0.3-<1.05	39	<0.06-<0.4	<0.06-<0.4	92 ± 6
1,7-Dimethylxanthine	<60	<60	164	<12-<24	<12-<24	154 ± 42
10-hydroxy-amitriptyline	<0.15	<0.15	102	<0.03-<0.06	<0.03-<0.06	98 ± 17
2-Hydroxy-ibuprofen	<80	<80	111	<16-<32	<16-<32	164 ± 41
4-Epianhydrochlortetracycline	<60	<60	35	<12-<24	<12-<24	15 ± 8
4-Epianhydrotetracycline [EATC]	<15	<15	88	<3-<6	<3-<6	81 ± 28
4-Epichlortetracycline [ECTC]	<15	<15	58	<3-<6	<3-<6	84 ± 12
4-Epioxytetracycline [EOTC]	<6	<6	107	<1.2-<2.4	<1.2-<2.4	80 ± 15
4-Epitetracycline [ETC]	<6	<6	114	<1.2-<2.4	<1.2-<2.4	111 ± 15
Diphenhydramine	<0.6	<0.6	98	<0.12-<0.24	<0.12-<0.24	89 ± 7
Doxorubicin	<24	<24	83	<4.8-<8	<4.8-<8	95 ± 4
Doxycycline	<6	<6	93	<1.2-<2.61	<1.2-<2.61	94 ± 19
Drospirenone	<8	<8	95	<1.6-<2.67	<1.6-<2.67	104 ± 9
Enrofloxacin	<3	<3	114	<0.6-<1.2	<0.6-<1.2	94 ± 18
Erythromycin-H2O	<2.3-2.52	<2.3-2.52	105	<0.46-<0.92	<0.46-<0.92	96 ± 4
Etoposide	<2-<3.35	<2-<3.35	106	<0.4-<2.48	<0.4-<2.48	101 ± 11
Flumequine	<1.5	<1.5	104	<0.3-<0.6	<0.3-<0.6	90 ± 10
Fluocinonide	<6	<6	88	<1.2-<2.4	<1.2-<2.4	88 ± 18
Fluoxetine	<1.5	<1.5	105	<0.3-<0.6	<0.3-<0.6	112 ± 5
Fluticasone propionate	<2	<2	64	<0.4-<0.8	<0.4-<0.8	38 ± 21
Furosemide	<40	<40	99	<8-<16	<8-<16	100 ± 20
Gemfibrozil	<1.5	<1.5	96	<0.3-<0.6	<0.3-<0.6	101 ± 4
Glipizide	<6	<6	92	<1.2-<2.4	<1.2-<2.4	99 ± 8
Glyburide	<3	<3	99	<0.6-<1.2	<0.6-<1.2	102 ± 16
Hydrochlorothiazide	<12-<12	<12-<20	73	<4-<4.8	<4-<4.8	36 ± 7
Hydrocortisone (Cortisol)[3]	<60	<60	106	<12-<24	<12-<24	101 ± 16
Ibuprofen	<15	<15	101	<3-<6	<3-<6	95 ± 4
Iopamidol	<80-<109	<80-<109	97	<16-<26.7	<16-<26.7	100 ± 8
Isochlortetracycline [ICTC]	<6	<6	125	<1.2-<2.4	<1.2-<2.4	59 ± 13
Lincomycin	<3	<3	38	<0.6-<1.2	<0.6-<1.2	112 ± 18
Lomefloxacin	<3	<3	127	<0.6-<1.2	<0.6-<1.2	97 ± 12
Medroxyprogesterone Acetate	<4	<4	96	<0.8-<1.33	<0.8-<1.33	105 ± 11
Melphalan	<24-<207	<24-<207	92	<4.8-<30.9	<4.8-<30.9	99 ± 10
Meprobamate	<4	<4	100	<2.67-<1.6	<2.67-<1.6	76 ± 3
Methylprednisolone	<4	<4	109	<0.8-<1.6	<0.8-<1.6	111 ± 13
Metoprolol	<1.5	<1.5	100	<0.3-<0.6	<0.3-<0.6	99 ± 6
Metronidazole	<4.4-<4.4	<4.4-<8.8	97	<1.03-<1.46	<1.03-<1.46	106 ± 3
Miconazole	<1.5	<1.5	91	<0.3-<0.6	<0.3-<0.6	61 ± 36
Minocycline	<60-<63	<60-<63	58	<12-<24	<12-<24	78 ± 28
Moxifloxacin	<4-<4.94	<4-<4.94	81	<0.8-<1.33	<0.8-<1.33	69 ± 43
Naproxen	<3	<3	111	<0.6-<1.2	<0.6-<1.2	95 ± 9

Norfloxacin	<15	<15	115	<3-<6	<3-<6	115 ± 6
Norfluoxetine	<1.5	<1.5	99	<0.365-<0.6	<0.365-<0.6	92 ± 5
Norgestimate	<3	<3	79	<0.6-<1.2	<0.6-<1.2	59 ± 40
Norverapamil	<0.15	<0.15	96	<0.03-<0.06	<0.03-<0.06	101 ± 17
Ofloxacin	<1.5	<1.5	121	<0.3-<0.6	<0.3-<0.6	108 ± 12
Ormetoprim	<0.6	<0.6	106	<0.12-<0.24	<0.12-<0.24	101 ± 6
Oxacillin	<3	<3	86	< 0.6-<1.2	< 0.6-<1.2	103 ± 34
Oxazepam	<4	<4	92	<0.8-<1.33	<0.8-<1.33	97 ± 5
Oxolinic Acid	<0.6	<0.6	130	<0.12-<0.24	<0.12-<0.24	92 ± 8
Oxytetracycline [OTC]	<6-<20	<6-<20	93	<1.2-<2.4	<1.2-<2.4	95 ± 15
Paroxetine	<4	<4	108	<0.8-<1.6	<0.8-<1.6	104 ± 9
Penicillin G	<3	<3	83	< 0.6-<1.2	< 0.6-<1.2	87 ± 19
Penicillin V	<3	<3	87	<0.6-<1.2	<0.6-<1.2	135 ± 20
Prednisolone	<6	<6	122	<1.2-<2.4	<1.2-<2.4	114 ± 27
Prednisone	<20	<20	106	<4-<8	<4-<8	114 ± 28
Promethazine	<0.4-<1.33	<0.4-<1.33	92	<0.266-<0.16	<0.266-<0.16	93 ± 9
Propoxyphene	<0.3	<0.3	106	<0.06-<0.12	<0.06-<0.12	99 ± 5
Propranolol	<2	<2	136	<0.4-<0.8	<0.4-<0.8	121 ± 11
Rosuvastatin	<4	<4	95	<0.8-<1.33	<0.8-<1.33	99 ± 9
Roxithromycin	<0.3	<0.3	72	<0.06-<0.12	<0.06-<0.12	102 ± 23
Sarafloxacin	<15	<15	122	<3-<6	<3-<6	105 ± 15
Sertraline	<0.4	<0.4	102	<0.08-<0.16	<0.08-<0.16	63 ± 31
Simvastatin	<20	<20	24	<4-<8	<4-<8	56 ± 29
Sulfachloropyridazine	<1.5	<1.5	120	<0.3-<0.6	<0.3-<0.6	106 ± 13
Sulfadiazine	<1.5	<1.5	104	<0.3-<0.6	<0.3-<0.6	117 ± 22
Sulfadimethoxine	<0.3	<0.3	100	<0.06-<0.12	<0.06-<0.12	89 ± 13
Sulfamerazine	<0.6	<0.6	97	<0.12-<0.24	<0.12-<0.24	98 ± 13
Sulfamethazine	<0.6	<0.6	91	<0.12-<0.24	<0.12-<0.24	101 ± 4
Sulfamethizole	<0.6	<0.6	109	<0.12-<0.24	<0.12-<0.24	90 ± 13
Sulfamethoxazole	<0.6	<0.6	104	<0.12-<0.24	<0.12-<0.24	99 ± 7
Sulfanilamide	<15-<150	<15-<150	34	<3-<6	<3-<6	72 ± 8
Sulfathiazole	<1.5	<1.5	111	<0.3-<0.6	<0.3-<0.6	109 ± 7
Tamoxifen	<0.4	<0.4	92	<0.08-<0.13	<0.08-<0.13	99 ± 11
Teniposide	<4	<4	30	<0.8-<3.01	<0.8-<3.01	58 ± 20
Tetracycline [TC]	<6-<20	<6-<20	85	<1.2-<8	<1.2-<8	94 ± 12
Theophylline	<60	<60	223	<12-<24	<12-<24	294 ± 41
Thiabendazole	<1.5	<1.5	114	<0.3-<0.6	<0.3-<0.6	104 ± 6
Trenbolone	<4	<4	125	<0.8-<1.6	<0.8-<1.6	92 ± 6
Trenbolone acetate	<0.3	<0.3	107	<0.06-<0.12	<0.06-<0.12	57 ± 11
Triclocarban	<3	<3	101	<0.6-<1.2	<0.6-<1.2	102 ± 13
Triclosan	<60	<60	115	<12-<24	<12-<24	102 ± 15
Trimethoprim	<1.5	<1.5	101	<0.3-<0.6	<0.3-<0.6	102 ± 5

Tylosin	<6	<6	80	<1.2-<2.4	<1.2-<2.4	88 ± 5
Valsartan	<4	<4	92	<0.8-<1.6	<0.8-<1.6	58 ± 29
Venlafaxine	<0.4	<0.4	89	0.16-<0.13	0.16-<0.13	100 ± 14
Verapamil	<0.15	<0.15	100	<0.03-<0.06	<0.03-<0.06	120 ± 28
Virginiamycin M1	<3	<3	57	<0.6-<1.2	<0.6-<1.2	90 ± 4
Warfarin	<1.5	<1.5	109	<0.3-<0.6	<0.3-<0.6	106 ± 10
Zidovudine	<24	<24	89	<4.8-<8	<4.8-<8	104 ± 12

[1] The analytes were from SGS AXYS Lists 1, 2, 3, 5 and 6 and involved 5 LC-MS/MS analytical runs from one extraction

[2]DL assumes sample size of 1 L for water and 2.5 g for plasma

[3]Hydrocortisone (cortisol) was determined but not reported because it was assumed to be present in all fish a natural hormone

Table S3. Water chemistry parameters for Jordan Harbour (JH), Cootes Paradise central marsh (CPM3), and Desjardins Canal outlet (all units are mg/L except chlorophyll a, µg/L)

Parameter	JH	JH	JH	JH	JH	CPM3	CPM3	CPM3	CPM3	CPM3	Canal outlet	Canal outlet	Canal outlet	Canal outlet	Canal outlet
Sample →	01-21-2014	01-24-2014	01-29-2014	02-03-2014	07-16-2014	01-21-2014	01-24-2014	01-29-2014	02-03-2014	07-16-2014	01-21-2014	01-24-2014	01-29-2014	02-03-2014	07-16-2014
pH					7.15					7.70					7.39
DO					8.6					6.3					14.2
Conductivity (uS/cm)					441					810					1066
NO_3NO_2-Filtered	1.62	1.25	1.94	0.555	0.013	1.54	2.55	2.52	2.58	0.04	13.1	14.1	14.4	14.3	14.3
NO_3NO_2-Unfiltered	0.922	1.25	2.1	0.556	0.014	1.54	2.48	2.55	2.59	0.038	13.4	14.4	14.4	14.3	14.3
NH_3-N-Filtered	0.048	0.053	0.051	0.025	0.064	0.039	0.061	0.105	0.112	0.011	0.208	0.392	0.418	1.37	0.101
NH_3-N-Unfiltered	0.044	0.059	0.035	0.023	0.167	0.034	0.104	0.064	0.113	0.035	0.194	0.388	0.417	1.41	0.182
ALK $CACO_3$	202	114	237	102	117	265	270	269	249	143	146	131	124	119	116
F-Filtered	0.23	0.14	0.26	0.12	0.17	0.21	0.27	0.27	0.27	0.28	0.47	0.5	0.53	0.53	0.51
CL-Filtered	98.7	77.7	114	38.7	35.1	118	137	134	230	110	331	267	281	290	190
SO4-Filtered	67.2	42.6	79	35.5	68.7	55.9	77.6	77.3	79.9	74.2	90.6	87.4	84	79.2	75.9
CA-Filtered	79.7	48.7	90.6	40.8	52.7	93	103	102	96.9	55.2	81.3	76.9	71.8	67.3	67.7
K-Filtered	5.7	4.52	6.33	2.11	3.2	4.99	6.58	6.72	6.61	5.26	10.3	11	11.2	11.2	9.98
MG-Filtered	23.4	12.3	27.7	10.6	14.8	29.1	31.1	30.9	28.8	25.1	21.4	20.7	19.5	18.2	18.3
NA-Filtered	58	45.2	64.7	23.3	23.1	66.9	79	77.9	136	62.5	198	161	170	178	119
SIO_2-Filtered	6.66	6.26	7.39	1.59	0.77	7.51	7.93	8.18	7.62	3.36	5.88	5.54	5.06	4.71	0.17
CHLA (µg/L)	< 0.1	0.1	18.7	< 0.1	38.3	< 0.1	< 0.1	27.1	67	6.2	< 0.1	< 0.1	NA	0.6	103
DOC	4.6	6.4	7.5	2.6	6.4	6.4	6.2	5.6	4.9	6.6	5.5	10.6	6.8	6.9	5.2
DIC	21.2	27.3	56.1	22.8	26	62	63	63	57.9	32.9	34.1	30.4	29	28.1	26.1
NO_2-N-Filtered	0.029	0.011	0.035	0.005	0.001	0.078	0.36	0.142	0.055	0.007	1.7	0.71	1.4	0.67	0.052
NO_2-N-Unfiltered	0.012	0.017	0.013	0.005	0.002	0.064	0.075	0.3	0.039	0.007	0.84	0.025	0.46	0.445	0.053
POC	0.245	0.323	1.88	0.155	2.33	0.198	0.31	1.16	1.41	0.566	0.593	0.65	0.7	0.578	2.32
PON	0.03	0.045	0.105	0.012	0.416	0.014	0.028	0.098	0.098	0.084	0.08	0.104	0.098	0.08	0.403
SRP-P-Filtered	0.028	0.077	0.016	0.012	0.036	0.013	0.015	0.013	0.018	0.001	0.087	0.102	0.091	0.163	0.007

SRP-P-Unfiltered	0.057	0.083	0.016	0.013	0.053	0.013	0.017	0.018	0.022	0.005	0.101	0.123	0.114	0.191	0.024
TKN-N-Filtered	0.432	0.516	0.492	0.234	0.564	0.49	0.643	0.584	0.658	0.47	1.08	1.48	1.6	2.48	0.963
TKN-N-Unfiltered	0.429	0.566	0.623	0.232	1.08	0.585	0.616	0.584	0.699	0.566	1.18	1.5	1.75	2.61	1.2
TN-N-Filtered	1.97	1.71	2.3	0.802	0.532	1.96	2.91	2.95	2.97	0.443	14.4	15.8	16.3	17.4	14
TN-N-Unfiltered	1.35	1.9	3.02	0.812	1.11	2.04	3.16	3.06	3.25	0.543	15.1	16.4	16.5	17.5	14.4
TP-P-Filtered	0.040	0.086	0.025	0.017	0.0567	0.019	0.023	0.022	0.025	0.011	0.107	0.125	0.12	0.194	0.028
TP-P-Unfiltered	0.075	0.105	0.210	0.020	0.156	0.024	0.039	0.034	0.058	0.032	0.137	0.165	0.195	0.285	0.128

Table S4. PPCPs (ng/g wet wt) in pooled plasma of caged and in individual plasma samples of wild goldfish from Cootes Paradise Marsh (CPM) and Jordan Harbour (JH) reference site. Site locations are shown in Figure S1 and S2.

	CPM1 Caged 07 2014	CPM2 Caged 07-2014	CPM3 caged 07-2014	JH caged 07-2014	CPM wild-01 2012	CPM wild-02 2012	CPM wild-03 2012
Sample Size (g, wet)	2.56	2.57	2.44	2.50	2.55	2.46	2.40
UNITS (wet weight)	ng/g	ng/g	ng/g	ng/g	ng/g	ng/g	ng/g
1,7-Dimethylxanthine	<11.7	<11.7	<12.3	<12	<11.8	<12.2	<12.5
10-hydroxy-amitriptyline	0.033	0.039	0.015	<0.03	0.015	0.036	0.058
2-Hydroxy-ibuprofen	<15.6	<15.6	<16.4	<16	<15.7	<16.3	<16.7
4-Epianhydrochlor-tetracycline	<11.7	<11.7	<12.3	<12	<11.8	<12.2	<12.5
4-Epianhydrotetracycline	<2.93	<2.92	<3.07	<3.0	<2.94	<3.05	<3.13
4-Epichlortetracycline	<2.93	<2.92	<3.07	<3.0	<2.94	<3.05	<3.13
4-Epioxytetracycline	<1.17	<1.17	<1.23	<1.2	<1.18	<1.22	<1.25
4-Epitetracycline	<1.17	<1.17	<1.23	<1.2	<1.18	<1.22	<1.25
Acetaminophen	<2.93	<2.92	<3.07	<3	<2.94	<3.05	<3.13
Alprazolam	<0.0586	<0.0584	<0.0615	<0.06	<0.0588	<0.061	<0.0625
Amitriptyline	0.029	0.029	0.031	<0.06	0.029	0.082	0.091
Amlodipine	<0.293	<0.356	<0.307	<0.3	<0.47	<0.305	<0.313
Amsacrine	<0.0156	<0.0156	<0.0182	<0.016	<0.0157	<0.0163	<0.0193
Anhydrochlortetracycline	2.97	<2.92	<3.07	<3.0	<2.94	<3.18	<3.13
Anhydrotetracycline	<2.93	<2.92	<3.07	<3.0	<2.94	<3.05	<3.13
Azathioprine	<0.391	<0.389	<0.41	<0.4	<0.392	<0.407	<0.417
Azithromycin	<0.293	<0.292	<0.307	<0.3	<0.294	<0.305	<0.313
Benzoylecgonine	<0.0586	<0.0584	<0.0615	<0.06	<0.0588	<0.061	<0.0625
Benztropine	<0.0977	<0.0973	<0.102	<0.1	<0.098	<0.102	<0.104
Betamethasone	<0.396	<0.374	<0.327	<0.374	<0.478	<0.621	<0.445
Bisphenol A	<97.7	<97.3	<102	<100	<98	<102	<104
Busulfan	<0.781	<0.778	<0.82	<0.8	<0.923	<0.91	<0.833
Caffeine	3.38	1.46	1.535	<3.0	1.47	1.525	1.565
Carbadox	<0.293	<0.292	<0.307	<0.3	<0.294	<0.305	<0.313
Carbamazepine	<0.293	<0.292	<0.307	<0.3	<0.294	<0.305	<0.313
Cefotaxime	NQ	NQ	NQ	NQ	NQ	NQ	NQ
Chlortetracycline [CTC]	<1.17	<1.17	<1.23	<1.2	<1.18	<1.22	<1.25
Ciprofloxacin	<1.17	<1.17	<1.23	<1.2	<1.27	<1.22	<1.25
Citalopram	0.1455	0.1625	0.041	<0.08	0.0392	0.127	0.13
Clarithromycin	<0.293	<0.292	<0.307	<0.3	<0.294	<0.305	<0.313
Clinafloxacin	<1.17	<1.17	<1.23	<1.2	<1.18	<1.22	<1.25
Clotrimazole	<0.0781	<0.0778	<0.082	<0.08	<0.0784	<0.0813	<0.0833
Cloxacillin	<0.586	<0.584	<0.615	<0.6	<0.588	<0.61	<0.625
Cocaine	<0.0293	<0.0292	<0.0307	<0.03	<0.0294	<0.0305	<0.0313

Colchicine	<0.156	<0.156	<0.164	<0.16	<0.157	<0.163	<0.167
Cyclophosphamide	<0.156	<0.156	<0.164	<0.16	<0.157	<0.163	<0.167
Daunorubicin	<1.56	<1.56	<1.64	<1.6	<1.57	<1.63	<1.67
DEET	0.416	0.41	0.314	0.235	0.234	0.584	0.56
Dehydronifedipine	<0.117	<0.117	<0.123	<0.12	<0.118	<0.122	<0.125
Demeclocycline	<2.93	<2.92	<3.07	<3.0	<2.94	<3.05	<3.13
Desmethyldiltiazem	<0.0293	<0.0292	<0.0307	<0.03	<0.0294	<0.0305	<0.0313
Diatrizoic acid	<4.69	<4.67	<4.92	<4.8	<20.3	<4.88	<5
Diazepam	<0.0586	<0.0584	<0.0615	<0.06	<0.0588	<0.061	<0.0625
Digoxigenin	<1.17	<1.17	<1.23	<1.2	<1.18	<1.22	<1.25
Digoxin	<1.17	<1.17	<1.23	<1.2	<1.18	<1.22	<1.25
Diltiazem	<0.0586	<0.0584	<0.0615	<0.06	<0.0588	<0.061	<0.0625
Diphenhydramine	0.23	0.213	0.062	<0.12	0.059	0.13	0.251
Doxorubicin	<4.69	<4.67	<4.92	<4.8	<4.71	<4.88	<5
Doxycycline	<1.17	<1.17	<1.23	<1.2	<1.18	<1.22	<1.25
Drospirenone	<1.56	<1.56	<1.64	<1.6	<1.57	<1.63	<1.67
Enrofloxacin	<0.586	<0.584	<0.615	<0.6	<0.588	<0.61	<0.625
Erythromycin-H2O	0.225	0.224	0.495	0.23	0.226	0.512	0.663
Etoposide	<1.02	<0.389	<0.525	<0.4	<0.392	<0.407	<0.417
Flumequine	1.1	0.146	0.344	<0.3	1.37	0.188	0.157
Fluocinonide	<1.17	<1.17	<1.23	<1.2	<1.18	<1.22	<1.25
Fluoxetine	0.302	0.146	0.154	<0.3	0.147	0.153	0.4
Fluticasone propionate	<0.391	<0.389	<0.41	<0.4	<0.392	<0.407	<0.417
Furosemide	<7.81	<7.78	<8.2	<8.0	<7.84	<8.13	<8.33
Gemfibrozil	0.147	0.146	0.154	<0.3	0.147	0.387	0.86
Glipizide	<1.17	<1.17	<1.23	<1.2	<1.18	<1.22	<1.25
Glyburide	<0.586	<0.584	<0.615	<0.6	<0.588	<0.61	<0.625
Hydrochlorothiazide	<3.91	<3.89	<4.1	<4.0	<3.92	<4.07	<4.17
Hydrocortisone	100	107	113	92.4	129	134	118
Ibuprofen	<2.97	<2.92	<3.07	<3.0	<2.94	<3.05	<3.13
Iopamidol	20.9	17.6	8.20	<16	7.85	8.15	8.35
Isochlortetracycline [ICTC]	<1.17	<1.17	<1.23	<1.2	<1.18	<1.22	<1.25
Lincomycin	<0.586	<0.584	<0.615	<0.6	<0.588	<0.61	<0.625
Lomefloxacin	<0.586	<0.584	<0.615	<0.6	<0.588	<0.61	<0.625
Medroxyprogesterone Acetate	<0.781	<0.778	<0.82	<0.8	<0.784	<0.813	<0.833
Melphalan	<4.69	<4.67	<4.92	<4.8	<4.71	<4.88	<5
Meprobamate	<2.6	<2.59	<2.73	<2.67	<2.61	<2.71	<2.78
Methylprednisolone	<0.781	<0.778	<0.82	<0.8	<0.784	<0.813	<0.833
Metoprolol	<0.297	<0.292	<0.307	<0.3	<0.328	<0.305	<0.313
Metronidazole	<0.781	<0.778	<0.874	<0.8	<0.914	<1.09	<1.26
Miconazole	<0.293	<0.292	<0.307	<0.3	<0.294	<0.305	<0.313
Minocycline	<11.7	<11.7	<12.3	<12	<11.8	<12.2	<12.5
Moxifloxacin	<0.781	<0.778	<0.82	<0.8	<0.784	<0.813	<0.833

Naproxen	<0.586	<0.584	<0.615	<0.6	<0.588	<0.61	<0.625
Norfloxacin	<2.93	<2.92	<3.07	<3.0	<2.94	<3.05	<3.13
Norfluoxetine	0.879	0.58	0.168	<0.413	0.300	0.654	1.1
Norgestimate	<0.586	<0.584	<0.615	<0.6	<0.588	<0.61	<0.625
Norverapamil	<0.0293	<0.0292	<0.0307	<0.03	<0.0294	<0.0305	<0.0313
Ofloxacin	<0.293	<0.292	<0.307	<0.3	<0.294	<0.305	<0.313
Ormetoprim	<0.117	<0.117	<0.123	<0.12	<0.118	<0.122	<0.125
Oxacillin	<0.586	<0.584	<0.615	<0.6	<0.588	<0.61	<0.625
Oxazepam	0.813	0.389	0.410	0.912	1.34	0.898	0.417
Oxolinic Acid	<0.117	<0.117	<0.123	<0.12	<0.118	<0.122	<0.125
Oxytetracycline [OTC]	<1.17	<1.17	<1.23	<1.2	<1.18	<1.22	<1.25
Paroxetine	<0.781	<0.778	<0.82	<0.8	<0.784	<0.813	<0.833
Penicillin G	<0.586	<0.584	<0.615	<0.6	<0.588	<0.61	<0.625
Penicillin V	<0.586	<0.584	<0.615	<0.6	<0.588	<0.61	<0.625
Prednisolone	<1.17	<1.29	<1.77	<1.2	<2.05	<1.47	<1.6
Prednisone	<3.91	<3.89	<4.1	<4.0	<3.92	<4.07	<4.17
Promethazine	<0.26	<0.259	<0.273	<0.266	<0.261	<0.27	<0.277
Propoxyphene	<0.0586	<0.0584	<0.0615	<0.06	<0.0588	<0.061	<0.0625
Propranolol	<0.391	<0.389	<0.41	<0.4	<0.392	<0.407	<0.417
Rosuvastatin	<0.781	<0.778	<0.82	<0.8	<0.784	<0.813	<0.833
Roxithromycin	<0.0586	<0.0584	<0.0615	<0.06	<0.0588	<0.061	<0.0625
Sarafloxacin	<2.93	<2.92	<3.07	<3.0	<2.94	<3.05	<3.13
Sertraline	0.105	0.039	0.041	<0.08	0.039	0.11	0.236
Simvastatin	<3.91	<3.89	<4.1	<4.0	<3.92	<4.07	<4.17
Sulfachloropyridazine	<0.293	<0.292	<0.307	<0.3	<0.294	<0.305	<0.313
Sulfadiazine	<0.293	<0.292	<0.307	<0.3	<0.294	<0.305	<0.313
Sulfadimethoxine	<0.0586	<0.0584	<0.0615	<0.06	<0.0588	<0.061	<0.0625
Sulfamerazine	<0.117	<0.117	<0.123	<0.12	<0.118	<0.122	<0.125
Sulfamethazine	<0.244	<0.165	<0.123	<0.12	0.166	0.084	0.066
Sulfamethizole	<0.117	<0.117	<0.123	<0.12	<0.118	<0.122	<0.125
Sulfamethoxazole	<0.117	<0.117	<0.123	<0.12	<0.118	<0.122	<0.125
Sulfanilamide	<2.93	<2.92	<3.07	<3.0	<2.94	<3.05	<3.13
Sulfathiazole	<0.293	<0.292	<0.307	<0.3	<0.294	<0.305	<0.313
Tamoxifen	<0.0781	<0.0778	<0.082	<0.08	<0.0784	<0.0813	<0.0833
Teniposide	<0.781	<0.778	<0.948	<0.8	<0.888	<1.29	<0.833
Tetracycline [TC]	<1.17	<1.17	<1.23	<1.2	<1.18	<1.22	<1.25
Theophylline	<11.7	<11.7	<12.3	<12	<11.8	<12.2	<12.5
Thiabendazole	<0.293	<0.292	<0.307	<0.3	<0.294	<0.305	<0.313
Trenbolone	<0.781	<0.778	<0.82	<0.8	<0.784	<0.813	<0.833
Trenbolone acetate	<0.0586	<0.0584	<0.0615	<0.06	<0.0588	<0.061	<0.0625
Triclocarban	<0.586	<0.584	<0.615	<0.6	<0.588	<0.61	<0.625
Triclosan	<11.7	<11.7	<12.3	<12	<11.8	<12.2	<12.5
Trimethoprim	<0.293	<0.292	<0.307	<0.3	<0.294	<0.305	<0.313

Tylosin	<1.17	<1.17	<1.23	<1.2	<1.18	<1.22	<1.25
Valsartan	<0.781	<0.778	<0.82	<0.8	<0.784	<0.813	<0.833
Venlafaxine	0.165	0.039	0.041	<0.08	0.039	0.258	0.042
Verapamil	<0.0293	<0.0292	<0.0307	<0.03	<0.0294	<0.0305	<0.0313
Virginiamycin M1	<0.586	<0.584	<0.615	<0.6	<0.588	<0.61	<0.625
Warfarin	<0.293	<0.292	<0.307	<0.3	<0.294	<0.305	<0.313
Zidovudine	<4.69	<4.67	<4.92	<4.8	<4.71	<4.88	<5
ΣAmitriptyline	0.06	0.07	0.05	<0.03	0.04	0.12	0.15
ΣIbuprofen	<3.0	<3.0	<3.0	<3.0	<3.0	<3.0	<3.0
ΣCaffeine	3.38	1.46	1.54	<3.0	1.47	1.53	1.57
ΣDiltiazem	<0.06	<0.06	<0.06	<0.06	<0.06	<0.06	<0.06
ΣDiazepam	0.81	0.39	0.41	0.91	1.34	0.90	0.42
ΣFluoxetine	1.18	0.73	0.32	0.00	0.45	0.81	1.50
Σcocaine	<0.03	<0.03	<0.03	<0.03	<0.03	<0.03	<0.03
Number detected[2]	16	15	15	3	16	16	16

[1]Cages deployed at CPM1 (Desjardin Canal), CPM2 (West Pond), CPM3 (Central marsh), CPM4 Jordan Harbour. See Map in Figure S1.

[2]Hydrocortisone (cortisol) was not included because it was detectable in all fish and assumed to be present as a natural hormone

Table S5. PPCPs (ng/g wet wt) in plasma of individual wild carp from Cootes Paradise Marsh (CPM-C) and in carp and brown bullhead from Jordan Harbour (JH-C and JH-BB)

	CPM-C 01 2011	CPM -C 20 2011	CPM-C 2 Sep 27, 2011	CPM-C 10 Sep 28, 2011	CPM-C 13 Sep 28, 2011	CPM-C 36 Oct 3, 2011	JH-C 75 Nov 7, 2011	JH-C 73 Nov 7, 2011	JH-C 82 Nov 7, 2011	JH-C 54 Nov 7, 2011	JH-C 74 Nov 7, 2011	JH-C 78 Nov 7, 2011	JH-BB
Sex	Male	Male	Male	Female	Female	Female	Female	Female	Female	Male	Male	Male	
Sample Size (g, wet)	2.37	2.32	3.48	3.31	3.21	3.50	3.32	2.43	2.49	2.36	2.35	2.40	2.63
UNITS (wet weight)	ng/g	ng/g	ng/g	ng/g	ng/g	ng/g	ng/g	ng/g	ng/g	ng/g	ng/g	ng/g	ng/g
1,7-Dimethylxanthine	<12.7	<12.9	<17.2	<18.1	<18.7	<17.1	<18.1	<12.3	<12	<12.7	<12.8	<12.5	<22.8
10-hydroxy-amitriptyline	0.032	0.016	0.053	0.024	0.047	0.025	<0.0953	<0.0309	<0.0301	<0.0318	<0.0319	<0.0313	<0.0614
2-Hydroxy-ibuprofen	<16.9	<17.2	<23	<24.2	<24.9	<22.9	<24.1	<16.5	17	<16.9	<17	<38.5	<30.4
4-Epianhydrochlor-tetracycline	<12.7	<12.9	<17.2	<18.1	<18.7	<17.1	<18.1	<12.3	<12	<12.7	<12.8	<12.5	<22.8
4-Epianhydro-tetracycline	<3.16	<3.23	<4.31	<4.53	<4.67	<4.29	<4.52	<3.09	<3.01	<3.18	<3.19	<3.13	<5.7
4-Epichlortetracycline	<3.16	<3.23	<4.31	<4.53	<4.67	<4.29	<4.52	<3.09	<3.01	<3.18	<3.19	<3.13	<5.7
4-Epioxytetracycline	<1.27	<1.29	<1.72	<1.81	<1.87	<1.71	<1.81	<1.23	<1.2	<1.27	<1.28	<1.25	<2.28
4-Epitetracycline	<1.27	<1.29	<1.72	<1.81	<1.87	<1.71	<1.81	<1.23	<1.2	<1.27	<1.28	<1.25	<2.28
Acetaminophen	<3.28	<3.23	<4.31	<4.53	<4.67	<4.29	<4.52	<3.09	<3.01	<3.18	<3.19	<8.33	<5.7
Alprazolam	<0.0633	<0.0647	<0.0931	<0.0906	<0.0935	<0.0857	<0.104	<0.0617	<0.0602	<0.0636	<0.0638	<0.0625	<0.114
Amitriptyline	<0.0633	<0.0647	<0.195	<0.0906	<0.129	<0.0857	<0.156	<0.0617	<0.0602	<0.0636	<0.0638	<0.0635	<0.129
Amlodipine	<0.316	<0.323	<0.444	<0.549	<0.467	<0.429	<0.452	<0.309	<0.301	<0.318	<0.319	<0.397	<0.57
Amsacrine	<0.0169	<0.0172	<0.0289	<0.0269	<0.0249	<0.0256	<0.0259	<0.0165	<0.0161	<0.017	<0.0184	<0.0348	<0.0304
Anhydrochlortetra-cycline	<3.16	<3.36	<4.31	<4.53	<4.67	<4.29	<4.52	<3.09	<3.01	<3.18	<3.19	<3.13	<5.7
Anhydrotetracycline	<3.16	<3.23	<4.31	<4.53	<4.67	<4.29	<4.52	<3.09	<3.01	<3.18	<3.19	<3.13	<5.7
Azathioprine	<0.422	<0.431	<0.61	<0.813	<0.711	<0.734	<0.603	<0.412	<0.402	<0.424	<0.426	<0.417	<0.97
Azithromycin	<0.316	<0.323	<0.431	<0.453	<0.467	<0.429	<0.452	<0.309	<0.301	<0.318	<0.319	<0.313	<0.57
Benzoylecgonine	<0.0633	<0.0647	<0.0862	<0.0921	<0.0935	<0.0857	<0.0904	<0.0617	<0.0602	<0.0636	<0.0638	<0.0625	<0.114
Benztropine	0.053	0.054	0.358	0.373	0.407	0.371	0.375	0.052	0.050	0.053	0.053	0.052	0.436
Betamethasone	<0.417	<0.53	<1.38	<0.453	<0.467	<0.429	<0.452	<0.309	<0.301	<0.392	<0.421	<0.331	<0.57
Bisphenol A	<105	<108	<144	<151	<156	<143	<151	<103	<100	<106	<106	<104	<190

Busulfan	<0.844	<0.862	<1.15	<1.21	<1.25	<1.14	<1.2	<0.823	<1	<0.847	<1.35	<1.38	<1.52
Caffeine	1.58	1.62	5.05	2.27	2.34	2.15	<4.52	<3.09	<3.01	<3.18	<3.19	<3.13	<5.7
Carbadox	<0.316	<0.323	<0.431	<0.453	<0.467	<0.429	<0.452	<0.309	<0.301	<0.318	<0.319	<0.313	<0.57
Carbamazepine	<0.316	<0.323	<0.431	<0.453	<0.467	<0.429	<0.452	<0.309	<0.301	<0.318	<0.319	<0.313	<0.57
Cefotaxime	NQ	NQ	<8.72	<8.54	<9.58	<5.87	<6.02	NQ	NQ	NQ	NQ	NQ	<7.6
Chlortetracycline [CTC]	<1.27	<1.29	<6.66	<6.74	<6.92	<6.32	<6.68	<1.23	<1.2	<1.27	<1.28	<1.25	<8.48
Ciprofloxacin	<1.27	<1.29	<1.72	<1.81	<1.87	<1.71	<1.81	<1.23	<1.2	<1.27	<1.28	<1.25	<2.28
Citalopram	<0.0844	<0.0862	<0.115	<0.121	<0.125	<0.114	<0.12	<0.0823	<0.0803	<0.0848	<0.0851	<0.0833	<0.152
Clarithromycin	<0.316	<0.323	<0.431	<0.453	<0.467	<0.429	<0.452	<0.309	<0.301	<0.318	<0.319	<0.313	<0.57
Clinafloxacin	<1.27	<1.29	<6.11	<6.39	<6.96	<6.28	<6.37	<1.23	<1.2	<1.27	<1.28	<1.84	<7.94
Clotrimazole	<0.0844	<0.0862	<0.115	<0.121	<0.125	<0.114	<0.12	<0.0823	<0.0803	<0.0848	<0.0851	<0.0833	<0.152
Cloxacillin	<0.633	<0.647	<0.862	<0.906	<0.935	<0.857	<0.904	<0.617	<0.602	<0.636	<0.638	<0.625	<1.14
Cocaine	<0.0317	<0.0323	<0.0431	<0.0453	<0.0467	<0.0429	<0.0452	<0.0309	<0.0301	<0.0318	<0.0319	<0.0313	<0.057
Colchicine	<0.169	<0.172	<0.519	<0.242	<0.249	<0.346	<0.334	<0.165	<0.161	<0.169	<0.17	<0.167	<0.425
Cyclophosphamide	<0.169	<0.172	<0.23	<0.242	<0.249	<0.229	<0.241	<0.165	<0.161	<0.169	<0.17	<0.167	<0.304
Daunorubicin	<1.69	<1.72	<2.3	<2.42	<2.49	<2.29	<2.41	<1.65	<1.61	<1.69	<1.7	<1.67	<3.04
DEET	0.194	0.35	1.09	1.15	1.1	0.743	0.914	0.083	0.865	0.331	0.494	1.09	2.59
Dehydronifedipine	<0.127	<0.129	<0.172	<0.181	<0.187	<0.171	<0.181	<0.123	<0.12	<0.127	<0.128	<0.125	<0.228
Demeclocycline	<3.16	<3.23	<4.31	<4.53	<4.67	<4.29	<4.52	<3.09	<3.01	<3.18	<3.19	<3.13	<5.7
Desmethyldiltiazem	<0.0317	<0.0323	<0.0431	<0.057	<0.0467	<0.0429	<0.0452	<0.0309	NQ	<0.0318	<0.0319	NQ	<0.057
Diatrizoic acid	<5.06	<5.17	<13.1	<7.25	<10.8	<10.8	<8.85	<4.94	<4.82	<5.42	<5.11	<6.01	<9.13
Diazepam	<0.0633	<0.0647	<0.0862	<0.0906	<0.27	<0.0857	<0.0904	<0.0617	<0.0602	<0.0636	<0.0638	<0.0625	<0.114
Digoxigenin	<1.27	<1.29	<2.23	<1.81	<1.87	<1.71	<1.81	<1.23	<1.2	<1.27	<1.28	<1.25	<2.28
Digoxin	<1.27	<1.29	<1.72	<1.81	<1.87	<1.71	<1.81	<1.23	<1.2	<1.27	<1.28	<1.25	<2.28
Diltiazem	<0.0716	<0.0647	<0.287	<0.302	<0.312	<0.286	<0.301	<0.0617	<0.0602	<0.0636	<0.0638	<0.0625	<0.38
Diphenhydramine	<0.127	<0.129	<0.172	<0.181	<0.187	<0.171	<0.181	<0.123	<0.12	<0.127	<0.128	<0.125	<0.228
Doxorubicin	<5.06	<5.17	<6.9	<7.25	<7.48	<6.86	<7.23	<4.94	<4.82	<5.08	<5.11	<5	<9.13
Doxycycline	<1.27	<1.29	<1.97	<1.88	<1.95	<1.72	<1.85	<1.23	<1.2	<1.27	<1.28	<1.25	<2.36
Drospirenone	<1.69	<1.72	<2.3	<2.42	<2.49	<2.29	<2.41	<1.65	<1.61	<1.69	<1.7	<1.67	<3.04
Enrofloxacin	<0.633	<0.647	<0.862	<0.906	<0.935	<0.857	<0.904	<0.617	<0.602	<0.636	<0.638	<0.625	<1.14
Erythromycin-H2O	0.52	0.54	0.676	0.348	0.359	0.791	0.347	0.583	0.488	0.684	0.727	0.507	<0.875

Etoposide	<0.422	<0.431	<0.575	<0.604	<0.623	<0.571	<0.602	NQ	<0.402	<0.424	<0.426	<1.63	<0.76
Flumequine	0.158	0.162	0.799	1.23	1.14	0.698	0.474	0.155	0.151	0.159	0.160	0.252	0.622
Fluocinonide	<1.27	<1.29	<1.72	<1.81	<1.87	<1.71	<1.81	<1.23	<1.2	<1.27	<1.28	<1.25	<2.28
Fluoxetine	<0.316	<0.323	<0.431	<0.453	<0.467	<0.429	<0.452	<0.309	<0.301	<0.318	<0.319	<0.313	<0.57
Fluticasone propionate	<0.422	<0.431	<0.575	<0.604	<0.623	<0.571	<0.602	<0.412	<0.402	<0.424	<0.426	<0.417	<0.76
Furosemide	<8.44	<8.62	<11.5	<12.1	<12.5	<11.4	<12	<8.23	<8.03	<8.47	<8.51	<8.33	<15.2
Gemfibrozil	0.158	0.162	0.724	0.227	0.234	0.827	<0.452	<0.309	<0.301	<0.318	<0.319	<0.433	<0.57
Glipizide	<1.27	<1.29	<1.72	<1.81	<1.87	<1.71	<1.81	<1.23	<1.2	<1.27	<1.28	<1.25	<2.28
Glyburide	<0.633	<0.647	<0.862	<0.906	<0.935	<0.857	<0.904	<0.617	<0.602	<0.636	<0.638	<0.625	<1.14
Hydrochlorothiazide	<4.22	<4.31	<3.45	<3.63	<3.74	<3.43	<3.61	<4.12	<4.02	<4.24	<4.26	<4.17	<4.56
Hydrocortisone[1]	343	210	1010	764	1010	875	1440	456	333	232	242	522	187
Ibuprofen	<3.16	<3.23	<4.31	<4.53	<4.67	4.67	<4.52	<3.09	<3.01	<3.18	<3.19	<3.13	<5.7
Iopamidol	<16.9	<17.2	<23	<24.2	<24.9	<22.9	<24.1	<16.5	<16.1	<17.5	<17	<26	<30.4
Isochlortetracycline	<1.27	<1.29	<1.72	<1.81	<1.87	<1.71	<1.81	<1.23	<1.2	<1.27	<1.28	<1.25	<2.28
Lincomycin	<0.633	<0.647	<0.862	<0.906	<0.935	<0.857	<0.904	<0.617	<0.602	<0.636	<0.638	<0.625	<1.14
Lomefloxacin	<0.633	<0.647	<0.862	<0.906	<0.935	<0.857	<0.904	<0.617	<0.602	<0.636	<0.638	<0.625	<1.14
Medroxyprogesterone Acetate	<0.844	<0.862	<1.15	<1.21	<1.25	<1.14	<1.2	<0.823	<0.803	<0.847	<0.851	<0.833	<1.52
Melphalan	<5.06	<5.17	<7.12	<7.25	<10.7	<6.86	<15	<4.94	<4.82	<5.08	<5.11	<5	<19.2
Meprobamate	<2.81	<2.87	<1.15	<1.21	<1.25	<1.14	<1.2	<2.74	<2.68	<2.82	<2.84	<2.78	<1.52
Methylprednisolone	<0.844	<0.862	<1.59	<2.59	<2.29	<1.8	<2.51	<0.823	<0.803	<0.847	<0.851	<0.833	<1.52
Metoprolol	<0.317	<0.372	<0.715	<0.564	<0.741	<0.541	<0.559	<0.309	<0.301	<0.318	<0.361	<0.417	<0.724
Metronidazole	<0.844	<1.24	<1.15	<1.21	<1.25	<1.14	<1.2	<0.823	<0.984	<0.867	<1.26	<1.63	<1.52
Miconazole	<0.316	<0.323	<0.431	<0.453	<0.467	<0.429	<0.452	<0.309	<0.301	<0.318	<0.319	<0.313	<0.57
Minocycline	<12.7	<12.9	<17.2	<18.1	<18.7	<17.1	<18.1	<12.3	<12	<12.7	<12.8	<12.5	<22.8
Moxifloxacin	<0.844	<0.862	<1.15	<1.21	<1.25	<1.14	<1.2	<0.823	<0.803	<0.847	<0.851	<0.833	<1.52
Naproxen	<0.633	<0.647	<0.862	<0.906	<0.935	<0.857	<0.904	<0.617	<0.602	<0.636	<0.638	<0.625	<1.14
Norfloxacin	<3.16	<3.23	<4.31	<4.53	<4.67	<4.29	<4.52	<3.09	<3.01	<3.18	<3.19	<3.13	<5.7
Norfluoxetine	0.338	0.179	0.216	0.227	0.234	0.215	<0.452	<0.315	<0.323	<0.321	<0.327	<0.517	<0.57
Norgestimate	<0.633	<0.647	<0.862	<0.906	<0.935	<0.892	<0.904	<0.617	<0.602	<0.636	<0.638	<0.625	<1.14
Norverapamil	<0.0317	<0.0323	<0.0431	<0.0453	<0.0467	<0.0429	<0.0452	<0.0309	<0.0301	<0.0318	<0.0319	<0.0313	<0.057

Ofloxacin	<0.316	<0.323	<0.431	<0.453	<0.467	<0.429	<0.452	<0.309	<0.301	<0.318	<0.319	<0.313	<0.57
Ormetoprim	<0.127	<0.129	<0.172	<0.181	<0.187	<0.171	<0.181	<0.123	<0.12	<0.127	<0.128	<0.125	<0.228
Oxacillin	<0.633	<0.647	<0.862	<0.906	<0.935	<0.857	<0.904	<0.617	<0.602	<0.636	<0.638	<0.625	<1.14
Oxazepam	1.18	3.6	33.7	4.81	9.4	7.4	8.7	2.57	2.27	0.977	0.991	0.912	<1.52
Oxolinic Acid	<0.127	<0.129	<0.172	<0.181	<0.187	<0.171	<0.181	<0.123	<0.12	<0.127	<0.128	<0.125	<0.228
Oxytetracycline [OTC]	<1.27	<1.29	<1.72	<1.81	<1.87	<1.71	<1.81	<1.23	<1.2	<1.27	<1.28	<1.25	<2.28
Paroxetine	<0.844	<0.862	<1.15	<1.21	<1.25	<1.14	<1.2	<0.823	<0.803	<0.847	<0.851	<0.833	<1.52
Penicillin G	<0.633	<0.647	<0.862	<0.906	<0.935	<0.857	<0.904	<0.617	<0.602	<0.636	<0.638	<0.625	<1.14
Penicillin V	<0.633	<0.647	<0.862	<0.906	<0.935	<0.857	<0.904	<0.617	<0.602	<0.636	<0.638	<0.625	<1.14
Prednisolone	<1.27	<1.43	<1.72	<1.81	<1.87	<1.71	<1.81	<1.33	<1.75	<1.29	<2.64	<4.26	<2.28
Prednisone	<4.22	<4.31	<9.59	<7.35	<8.12	<12.8	<6.02	<4.12	<4.02	<4.24	<4.26	<4.17	<8.26
Promethazine	<0.281	<0.287	<0.178	<0.121	<0.169	<0.114	<0.12	<0.274	NQ	<0.282	<0.283	NQ	<0.152
Propoxyphene	<0.0633	<0.0647	<0.0862	<0.0906	<0.0935	<0.0857	<0.0904	<0.0617	<0.0602	<0.0636	<0.0638	<0.0625	<0.114
Propranolol	<0.422	<0.431	<0.575	<0.604	<0.623	<0.571	<0.602	<0.412	<0.402	<0.424	<0.426	<0.417	<0.76
Rosuvastatin	<0.844	<0.862	<1.15	<1.21	<1.25	<1.14	<1.2	<0.823	<0.803	<0.847	<0.851	<0.833	<1.52
Roxithromycin	<0.0633	<0.0647	<0.0862	<0.0906	<0.0935	<0.0857	<0.0904	<0.0617	<0.0602	<0.0636	<0.0638	<0.0625	<0.114
Sarafloxacin	<3.16	<3.23	<4.31	<4.53	<4.67	<4.29	<4.52	<3.09	<3.01	<3.18	<3.19	<3.13	<5.7
Sertraline	0.092	0.043	0.058	0.061	0.063	0.125	<0.12	<0.0823	<0.0803	<0.0848	<0.0851	<0.0833	<0.152
Simvastatin	<4.22	<4.31	<5.75	<6.04	<6.23	<5.71	<6.02	<4.12	<4.02	<4.24	<4.26	<4.17	<7.6
Sulfachloropyridazine	<0.316	<0.323	<0.431	<0.453	<0.467	<0.429	<0.452	<0.309	<0.301	<0.318	<0.319	<0.313	<0.57
Sulfadiazine	<0.316	<0.323	<0.431	<0.453	<0.467	<0.429	<0.452	<0.309	<0.301	<0.318	<0.319	<0.313	<0.57
Sulfadimethoxine	<0.0633	<0.0647	<0.0862	<0.0906	<0.0935	<0.0857	<0.0904	<0.0708	<0.0602	<0.0636	<0.0638	<0.0625	<0.114
Sulfamerazine	<0.127	<0.129	<0.172	<0.181	<0.187	<0.171	<0.181	<0.123	<0.12	<0.127	<0.128	<0.125	<0.228
Sulfamethazine	<0.152	<0.131	<0.172	<0.181	<0.187	<0.171	<0.181	<0.152	<0.13	<0.194	<0.143	<0.145	<0.228
Sulfamethizole	<0.127	<0.129	<0.172	<0.181	<0.187	<0.171	<0.181	<0.123	<0.12	<0.127	<0.128	<0.125	<0.228
Sulfamethoxazole	<0.127	<0.129	<0.172	<0.181	<0.187	<0.171	<0.181	<0.123	<0.12	<0.127	<0.128	<0.125	<0.228
Sulfanilamide	<3.16	<3.23	<4.31	<4.53	<4.67	<4.29	<4.52	<3.09	<3.01	<3.18	<3.19	<3.13	<5.7
Sulfathiazole	<0.316	<0.323	<0.431	<0.453	<0.467	<0.429	<0.452	<0.309	<0.301	<0.318	<0.319	<0.313	<0.57
Tamoxifen	<0.0844	<0.0862	<0.115	<0.121	<0.125	<0.114	<0.12	<0.0823	<0.0803	<0.0848	<0.0851	<0.0833	<0.152
Teniposide	<0.844	<0.862	<1.15	<1.45	<1.67	<1.14	<1.2	NQ	NQ	<0.847	NQ	NQ	<1.52
Tetracycline [TC]	<1.27	<1.29	<5.75	<6.04	<6.23	<5.71	<6.02	<1.23	<1.2	<1.27	<1.28	<1.25	<7.6

Theophylline	<12.7	<12.9	<30.9	<26.1	<30.4	<23.2	<27.4	<12.3	<12	<12.7	<12.8	<12.5	<30.3
Thiabendazole	<0.316	<0.323	<0.431	<0.453	<0.467	<0.429	<0.452	<0.309	<0.301	<0.318	<0.319	<0.313	<0.57
Trenbolone	<0.844	<0.862	<1.15	<1.21	<1.25	<1.14	<1.2	<0.823	<0.803	<0.847	<0.851	<0.833	<1.52
Trenbolone acetate	<0.0633	<0.0647	<0.119	<0.199	<0.139	<0.126	<0.122	<0.0617	<0.0602	<0.0636	<0.0638	<0.0625	<0.114
Triclocarban	0.317	0.324	0.431	0.453	0.468	3.35	<0.904	<0.617	<0.602	<0.636	<0.638	<0.625	<1.14
Triclosan	<12.7	<12.9	<17.2	<18.1	<18.7	<17.1	<18.1	<12.3	<12	<12.7	<12.8	<12.5	<22.8
Trimethoprim	<0.316	<0.323	<0.431	<0.453	<0.467	<0.429	<0.452	<0.309	<0.301	<0.318	<0.319	<0.313	<0.57
Tylosin	<1.27	<1.29	<1.72	<1.81	<1.87	<1.71	<1.81	<1.23	<1.2	<1.27	<1.28	<1.25	<2.28
Valsartan	0.422	0.431	1.23	0.605	0.625	0.570	<1.2	<0.823	<0.803	<0.847	<0.851	<0.833	<1.52
Venlafaxine	0.042	0.268	0.086	0.061	0.063	0.057	0.060	0.041	0.040	0.042	0.043	0.436	<0.176
Verapamil	<0.0317	<0.0323	<0.0431	<0.0453	<0.0467	<0.0429	<0.0452	<0.0309	<0.0301	<0.0318	<0.0319	<0.0313	<0.057
Virginiamycin M1	<0.633	<0.647	<0.862	<0.906	<0.935	<0.857	<0.904	<0.617	<0.602	<0.636	<0.638	<0.625	<1.14
Warfarin	<0.316	<0.323	<0.431	<0.453	<0.467	<0.429	<0.452	<0.309	<0.301	<0.318	<0.319	<0.313	<0.57
Zidovudine	<5.06	<5.17	<6.9	<7.25	<7.48	<6.86	<7.23	<4.94	<4.82	<5.08	<5.11	<5	<9.13
ΣAmitriptyline	0.03	0.02	0.05	0.02	0.05	0.02	<0.06	<0.06	<0.06	<0.06	<0.06	<0.06	<0.06
ΣIbuprofen	<3.0	<3.0	<3.0	<3.0	<3.0	4.67	<3.0	<3.0	17.00	<3.0	<3.0	<3.0	<3.0
ΣCaffeine	1.58	1.62	5.05	2.27	2.34	2.15	<3.0	<3.0	<3.0	<3.0	<3.0	<3.0	<3.0
ΣDiltiazem	<0.06	<0.06	<0.06	<0.06	<0.06	<0.06	<0.06	<0.06	<0.06	<0.06	<0.06	<0.06	<0.06
ΣDiazepam	1.18	3.60	33.70	4.81	9.40	7.40	8.70	2.57	2.27	0.98	0.99	0.91	<0.06
ΣFluoxetine	0.34	0.18	0.22	0.23	0.23	0.21	<0.30	<0.30	<0.30	<0.30	<0.30	<0.30	<0.30
Σcocaine	<0.03	<0.03	<0.03	<0.03	<0.03	<0.03	<0.03	<0.03	<0.03	<0.03	<0.03	<0.03	<0.03
Number detected[1]	14	14	14	14	14	15	7	7	8	7	7	7	4

[1] Hydrocortisone (cortisol) was not included because it was detectable in all fish and assumed to be present as a natural hormone

Table S6. Comparison of average and maximum concentrations in surface water from the Cootes Paradise Marsh far field site (CPM3; N=3)) with results for rivers and streams reported in the review of global studies on pharmaceuticals by aus der Beek et al. [9]

PPCP	Global No of studies	Global average [1] (ng/L)	CPM3 average (ng/L)	CPM3 max (ng/L)	CPM avg > global	CPM max > global
Amitriptyline	2	14.8	0.65	0.95		
Azithromycin	22	209.1	3.04	7.57		
Carbadox	3	0.0	<DL			
Carbamazepine	122	264.9	22.06	52.9		
Cefotaxime	11	1.0	<DL	0.00		
Ciprofloxacin	65	36.3	7.97	9.50		
Citalopram	11	49.4	2.99	5.94		
Clarithromycin	28	21.4	9.92	26.1		x
Clinafloxacin	3	0.0	19.73	24.1	x	x
Clotrimazole	1	4.0	0.21	0.21		
Cloxacillin	4	0.0	<DL			
Cyclophosphamide	1	0.0	<DL			
Demeclocycline	4	7.5	<DL			
Diatrizoic acid	16	241.8	<DL			
Diazepam	37	0.7	<DL			
Digoxin	5	0.0	<DL			
Diltiazem	5	18.0	0.49	0.65		
Diphenhydramine	11	244.5	3.08	7.62		
Doxycycline	11	18.1	<DL			
Enrofloxacin	22	10.1	<DL			
Erythromycin-H2O	16	150.9	4.29	6.56		
Flumequine	21	4.5	<DL			
Fluoxetine	26	12.0	0.77	0.78		
Furosemide	16	31.3	<DL			
Gemfibrozil	35	120.5	0.77	0.78		
Hydrochlorothiazide	6	72.9	<DL			
Ibuprofen	135	96.3	<DL			
Iopamidol	42	119.1	540.67	768	x	x
Lincomycin	16	8.6	<DL			
Lomefloxacin	20	0.8	<DL			
Meprobamate	5	7.9	3.43	6.14		
Metoprolol	57	91.5	4.69	8.55		
Metronidazole	10	8.4	<DL			
Miconazole	1	2.0	<DL			
Minocycline	3	0.0	<DL			
Naproxen	76	57.7	30.56	85.0		x

Norfloxacin	43	46.5	<DL			
Norfluoxetine	11	0.4	<DL			
Ofloxacin	65	85.1	<DL			
Ormetoprim	9	0.0	<DL			
Oxacillin	5	0.0	<DL			
Oxazepam	1	11.4	5.76	10.8		
Oxolinic acid	11	4.1	<DL			
Paroxetine	18	12.5	<DL			
Propranolol	24	19.8	<DL			
Roxithromycin	66	69.9	<DL			
Sarafloxacin	11	0.4	<DL			
Sertraline	10	2.3	0.95	1.26		
Sulfachloropyridazine	30	86.2	<DL			
Sulfadiazine	40	37.8	<DL			
Sulfadimethoxine	31	71.9	0.31	0.46		
Sulfamerazine	15	14.4	<DL			
Sulfamethazine	50	34.4	0.30	0.30		
Sulfamethizole	10	2.5	<DL			
Sulfamethoxazole	147	106.4	8.78	19.8		
Sulfanilamide	5	31.6	<DL			
Sulfathiazole	30	12.2	<DL			
Tamoxifen	6	8.8	<DL			
Theophylline	3[2]	42.0	89.70	89.7	x	x
Thiabendazole	8	30.0	<DL			
Triclocarban	5	41.1	<DL			
Triclosan	33	25.6	<DL			
Trimethoprim	84	54.0	<DL			
Tylosin	24	15.3	<DL			
Valsartan	5	12.6	3.66	5.63		
Venlafaxine	27	107.6	7.99	14.9		
Zidovudine	1	5.9	<DL			
4-Epianhydrochlortetracy	3	0.0	<DL			
4-Epianhydrotetracycline	3	0.0	<DL			
4-Epichlortetracycline	3	0.0	<DL			
4-Epioxytetracycline	3	0.0	<DL			

[1]Average of the mean concentrations in rivers and streams reported by aus der Beek et al.[9] in their review of global studies on pharmaceutics.

[2]Average of 3 median values

Table S7. Comparison of PPCP concentrations in fish tissue reported in Meador et al. and in the present study (ng/g). Both studies used the same analytical laboratory and methodology.

Analytes common to Meador et al. (2016) and this study	Meador et al.	Meador et al.	This study	This study
	Salmon (Whole body)	Sculpin (Whole body)	Goldfish plasma	Carp plasma
Alprazolam		0.38		
Amitriptyline	0.58 - 0.68		0.03 -0.09	
Amlodipine	0.62 - 1.0			
Azithromycin	1.7			
Benzoylecgonine				
Benztropine	0.2			0.05 -0.41
Bisphenol A	3.3 - 41	3.6 - 4.5		
Caffeine	18	13	1.47 -1.57	1.58 -5.05
Carbamazepine				
Ciprofloxacin		17		
Clarithromycin				
Cocaine				
DEET	0.39 - 1.6	0.41 - 2.2	0.23 -0.58	0.19 -1.15
Dehydronifedipine				
Desmethyldiltiazem	0.06 - 1.5	0.07 - 0.08		
Diazepam	0.39	0.25		
Diltiazem	1.4 - 1.6			
Diphenhydramine	0.24 - 2.7	0.28	0.06 -0.25	
Fluocinonide	6.5			
Erythromycin-H2O	0.9		0.23 -0.66	0.35 -0.79
Fluoxetine	4.9		0.15 -0.4	
Furosemide				
Gemfibrozil	1.3		0.15 -0.86	0.16 -0.83
Glipizide				
Glyburide				
Hydrochlorothiazide				
Ibuprofen				4.67 -4.67
Lincomycin				
Meprobamate				
Metoprolol				
Miconazole	1.8			
Naproxen				
Norfluoxetine	0.68 - 3.2		0.17 -1.1	0.18 -0.34
Norverapamil	0.12 - 0.47	0.20 - 0.30		
Ofloxacin				
Ormetoprim	44 - 1600			

Paroxetine				
Promethazine				
Propoxyphene				
Propranolol				
Roxithromycin				
Sertraline	17	0.2	0.04 -0.24	0.04 -0.13
Simvastatin				
Sulfadiazine	0.88			
Sulfadimethoxine	0.34-17			
Sulfamerazine	0.51			
Sulfamethoxazole				
Thiabendazole				
Triclocarban	6.5			0.32 -3.35
Triclosan	26			
Trimethoprim				
Valsartan				0.42 -1.23
Verapamil	0.30 - 0.60	0.07 - 0.27		
Virginiamycin M1	10	8 - 34		
Warfarin				
10-hydroxy-amitriptyline	0.09	0.13	0.01 -0.06	0.02 -0.05
1,7-Dimethylxanthine				
2-Hydroxy-ibuprofen				

Table S8. Comparison of results for 18 PPCPs detected in fish plasma in this study with other recent reports for the same compounds in fish plasma. Results for other tissues are included for papers published post-2012. Earlier studies were reviewed by Huerta et al. [10]. All concentrations are ng/g wet weight or ng/mL for plasma (means ± standard deviations provided by the authors or calculated from supporting information)

Tissue	plasma	plasma	plasma	plasma	plasma	plasma	Liver & muscle	whole fish	whole fish	plasma	plasma
Common names	rainbow trout	rainbow trout	longear sunfish	carp	Carp[a] and tilapia	crucian carp & common carp	mullet	salmon	sculpin	goldfish	carp
Species >>>	Oncorhyn-chus mykiss	Oncorhyn-chus mykiss	Lepomis megalotis	Cyprinus carpio	Tilapia aurea, C. idellus and C. molitorella	Carassius carassius & Cyprinus carpio	Chelon aurata	Oncorhynchus tshawytscha	Leptocottus armatus		
PPCPs in this study	Brown et al. [11]	Fick et al. [12]	Du et al. [13]	Scott et al. [14]	Zhao et al. [15]	Tanoue et al.[16]	Moreno-González et al. [17]	Meador et al. [18]	Meador et al. [18]	This study	This study
Iopamidol	-	-	-	-	-	-	-	-	-	8.12	<mdl
ΣCaffeine	-	-	-	-	-	-	-	18	13	1.52	2.50
Sulfamethazine	-	-	-	-	6.47	-	-	-	-	0.11	<mdl
Flumequine	-	-	-	-	-	<mdl-1.7	-	-	-	0.57	0.70
DEET	-	-	-	-	-	-	-	0.39 - 1.6	0.41 - 2.2	0.46	0.77
ΣDiazepam	-	<LOQ-0.9	-	-	-	-	0.3 ± 0.3	0.39	0.25	0.88	10.02
Erythromycin-H_2O	-	-	-	-	29.4	-	-	0.9	<mdl	0.47	0.54
Valsartan	-	-	-	-	-	-	-	<mdl	<mdl	<mdl	0.65
Diphenhydramine	-	-	3.0±2.2	11	-	0.66±0.46	-	0.24 - 2.7	0.28	0.15	<mdl
Venlafaxine	-	-	-	-	-	-	0.68 ± 1.3	-	-	0.11	0.10
Citalopram	-	-	-	-	-	-	-	-	-	0.10	<mdl
ΣFluoxetine	-	-	-	-	-	-	-	4.9	<mdl	0.92	0.23
Benztropine	-	-	-	-	-	-	-	0.2	<mdl	<mdl	0.27
ΣIbuprofen	<mdl-84	<LOQ-1.2	-	-	-	-	-	<mdl	<mdl	<mdl	4.67
Gemfibrozil	<8-210	-	-	-	-	-	-	1.3	<mdl	0.46	0.39
Triclocarban	-	-	-	-	-	0.34±0.20	-	6.5	<mdl	<mdl	0.89
ΣAmitriptyline	-	-	-	-	-	-	-	0.58 - 0.68	<mdl	0.10	0.03
Sertraline	-	0.30-2.4	-	-	-	0.21±0.18	-	17	0.2	0.13	0.07

[a] grass carp (Ctenopharynodon idellus) and mud carp (Cirrhinus molitorella)

Table S9. Measured and predicted bioaccumulation factors (conc in fish plasma/conc in water; L/kg; BAF_P) for PPCPs detectable in fish from Cootes Paradise Marsh and Jordan Harbour. Whole body BAFs (BAF_{WB}) predicted for lower trophic level fish using the BCF-BAF model in EPISuite[19].

PPCP	BAF_P at CPM3		BAF_P at CPM3		BAF_P at JH		CPM1	CPM2	CPM3	JH		Predicted BAF	
	wild goldfish		carp		carp		goldfish	goldfish	goldfish	goldfish		Biotrans-formation	Lower trophic
	average	Range[1]	average	range	average	range	caged	caged	caged	caged	log Kow[2]	t1/2 (d)[3]	BAF_{WB}[4]
ΣAmitriptyline	115	49-165	36	18-59	334		24	9	198		4.92	0.46	418
Benztropine			671	131-1015	292	153-1148					3.85	0.34	233
ΣCaffeine	15	15-16	25	16-51			15	5	27		-0.07	0.02	1
Citalopram	44	18-58					4	6	52		3.74	0.53	191
DEET	9	4-11	15	4-22	32	4-51	4	4	4	9	2.18	0.16	9
ΣDiazepam	82	38-124	927	109-3120			18	5	198		2.24	0.60	37
Diphenhydramine	113	45-193					7	5	198		3.27	0.04	24
Erythromycin-H2O	71	34-101	82	53-121	148	102-214	36	4	201	86	3.06	0.02	12
Flumequine	261	68-626	319	72-562				38	277		1.60	0.06	3
ΣFluoxetine	906	310-1480	231	176-334			263	689	207		3.82	3.0	352
Gemfibrozil	598	190-1110	511	208-1088			189	27	198		4.14	2.0	1396
ΣIbuprofen			115	115-115							3.97	1.5	393
Iopamidol	38	37-40					75	50	13		-2.21	0.004	1
Sertraline	359	109-659	205	120-349			50	27	34		5.29	35	24210
Sulfamethazine	346	197-546									0.19	0.03	1
Triclocarban			586	208-2204							4.90	0.86	760
Valsartan			199	129-377							3.07	0.73	187
Venlafaxine	14	5-31	12	5-32	64	33-356	3		52		3.28	0.12	54

[1]Range based on range of concentrations in plasma of wild goldfish or carp (data in Table S3 and S4). Water concentrations were assumed to be constant ie average water concentrations during or near time of collection.

[2]Log octanol-water partition coefficient from the US EPA Chemistry Dashboard (https://comptox.epa.gov/dashboard)

[3]Predicted biotransformation half-life =log(2)/biotransformation rate (d^{-1}) for a 100 g fish from the BCF/BAF model in EPISuite Ver 4.1

[4]Predicted BAF_{WB} for lower trophic level fish using the BCF/BAF model in EPISuite Ver 4.1

Figure S1. Locations in Cootes Paradise Marsh for water sampling (CPM1, CPM2, CPM3) and for caged goldfish in (Hamilton ON). Location is shown with red square on the inset map. The base map is from the Atlas of Canada (with permission of Natural Resources Canada).

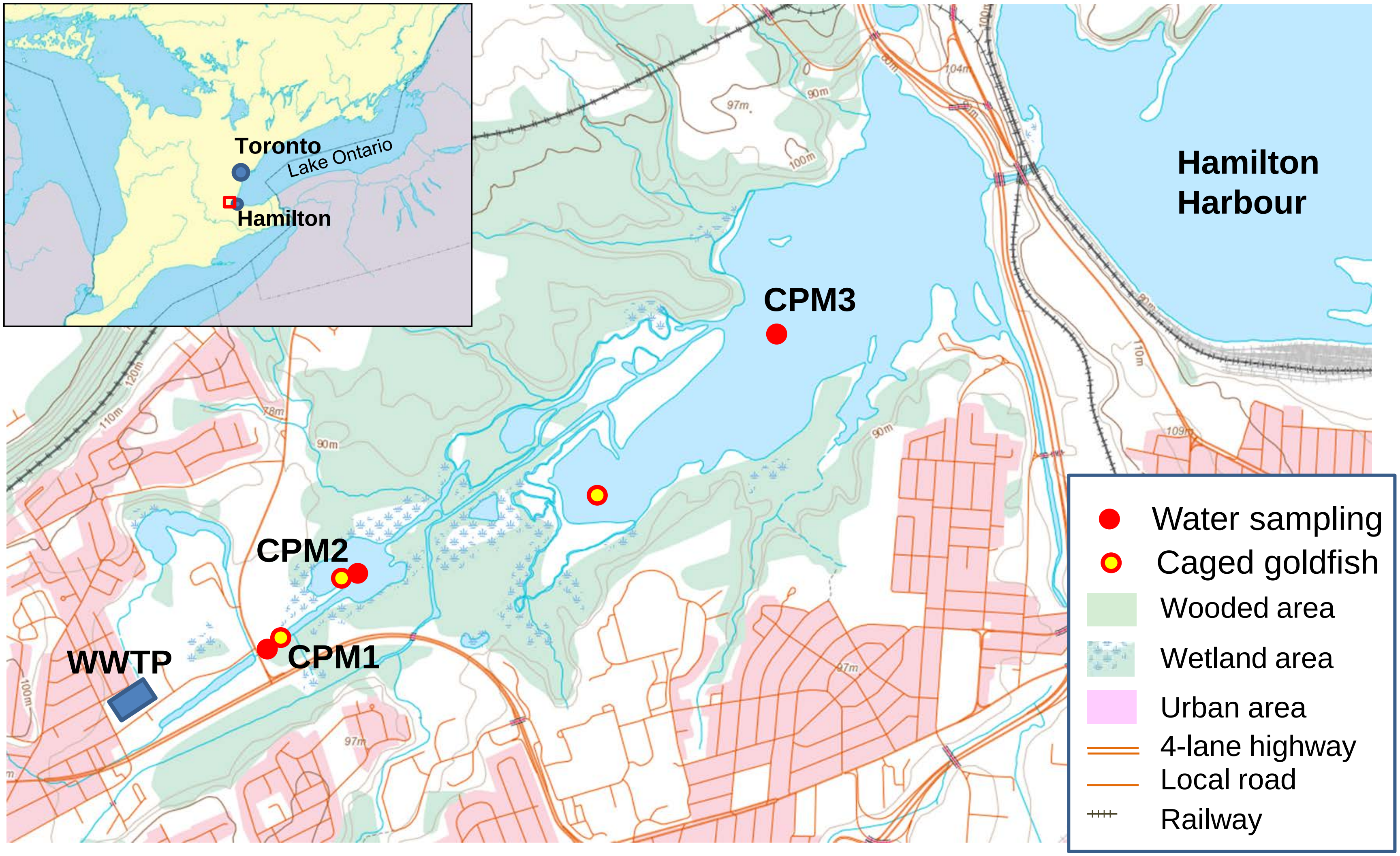

Figure S2. Locations for water sampling and for caged goldfish in Jordon Harbour (near Jordan ON). Location is shown with red square on the inset map. The base map is from the Atlas of Canada (with permission of Natural Resources Canada).

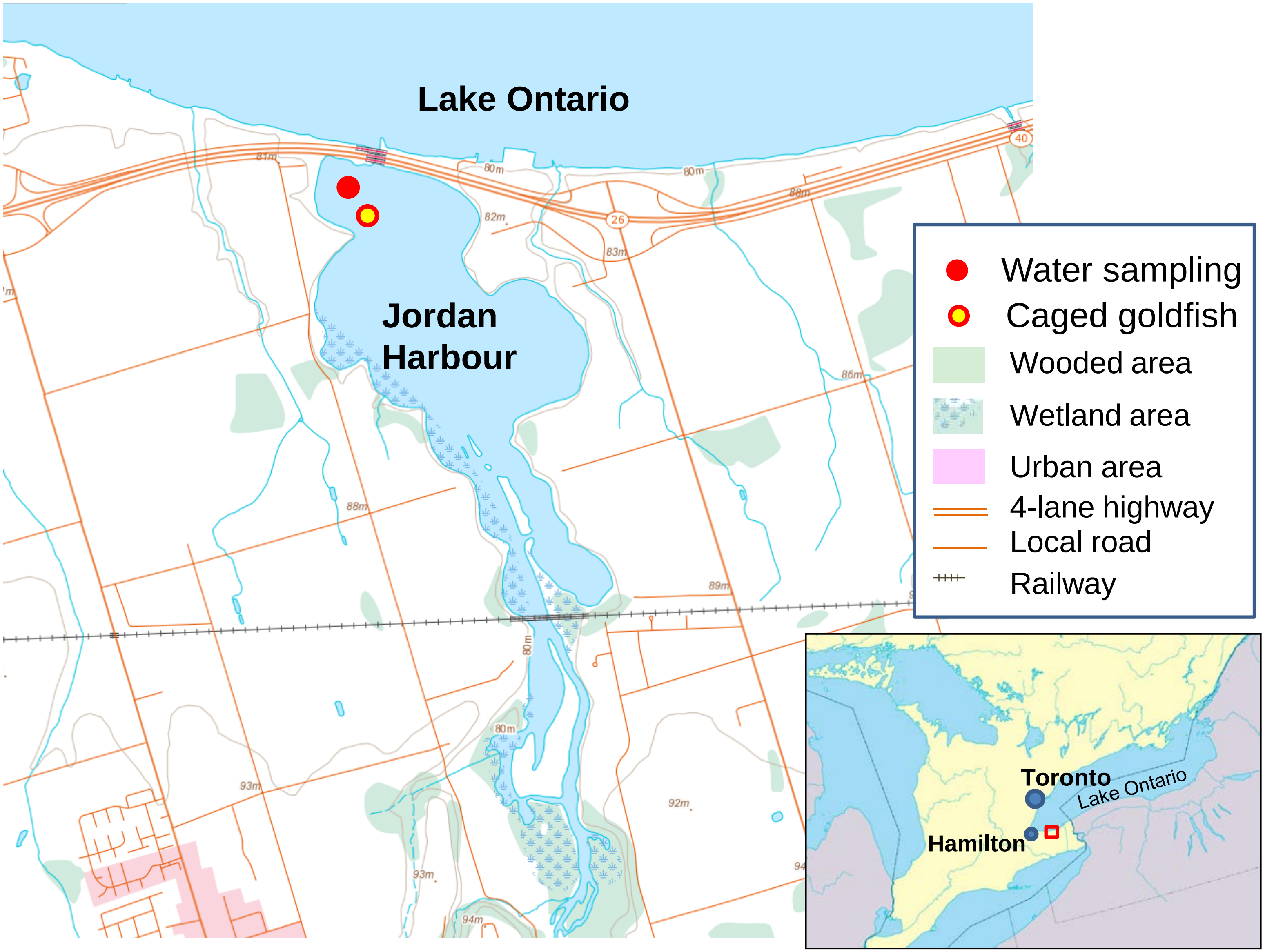

Figure S3. Mean concentrations and range of PPCPs detected in plasma of carp from Jordan Harbour, a reference site, and in a pooled sample of from caged goldfish collected in July 2014. See Figure 1 for the identities of the analytes on the horizontal axis.

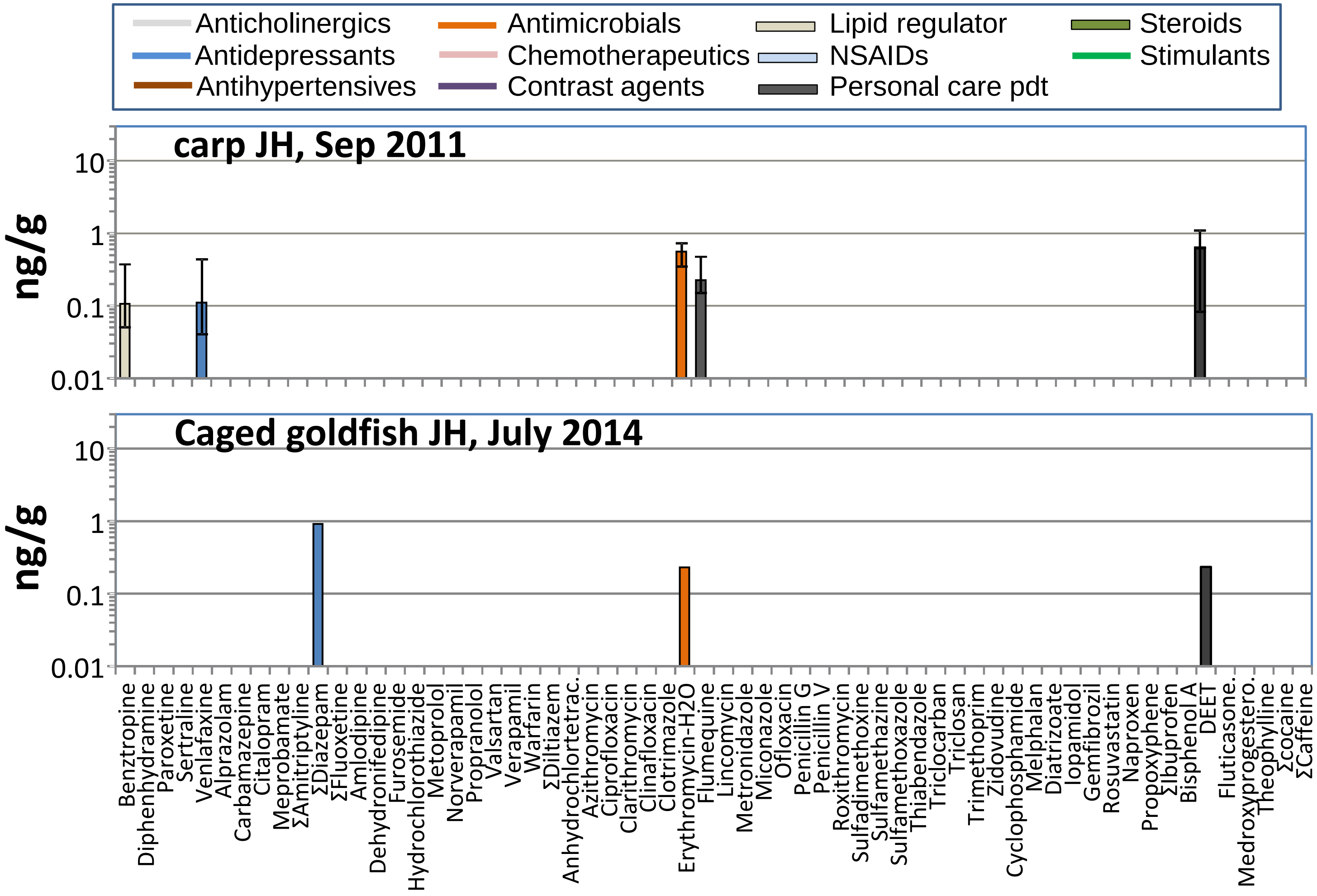

Figure S4. Bioaccumulation factors (L/kg) for PPCPs in caged goldfish from 3 sites in Cootes Paradise and in Jordan Harbour reference site (conc'n in fish plasma ÷ conc'n in water). Substances labelled with "<" had BAF_Ps that could not be calculated due to non-detect concentrations in water or fish

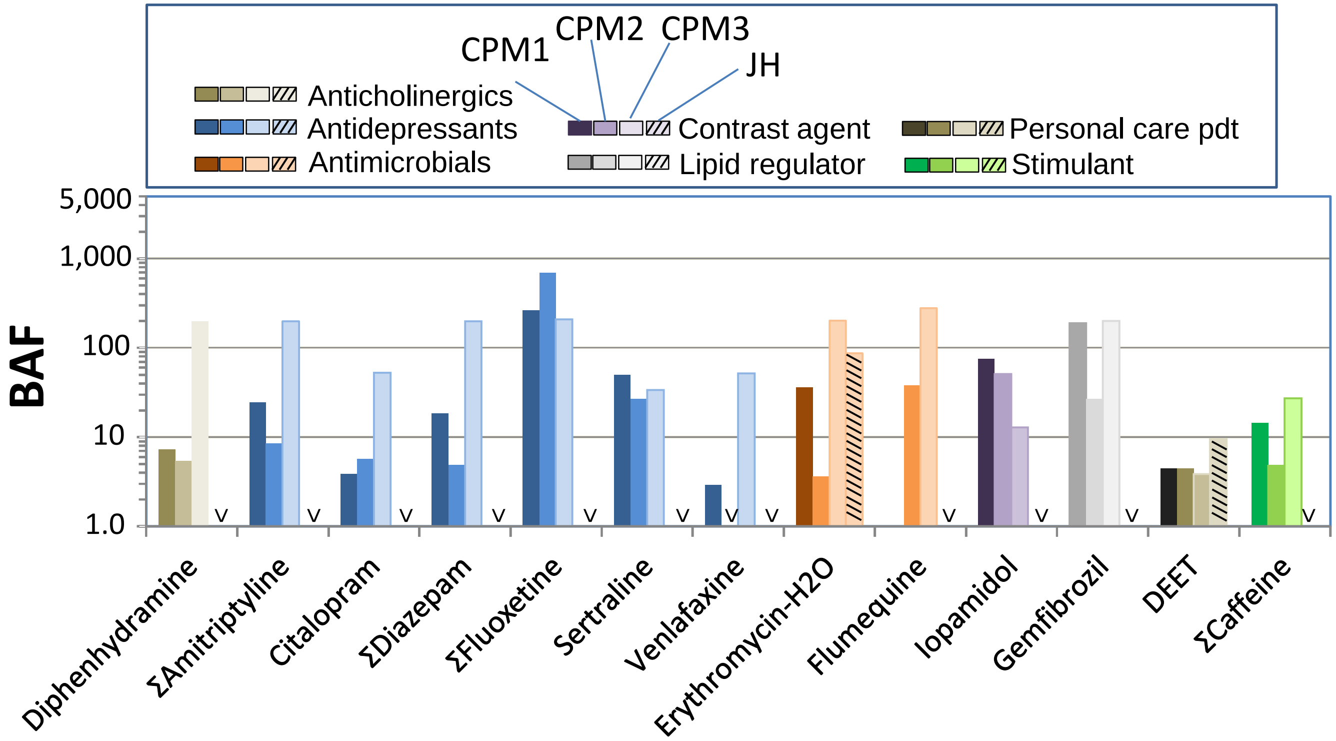

Figure S5. Measured plasma BAFs (BAF_P) for caged goldfish and water concentrations at 3 sites in Cootes Paradise Marsh (CPM). A statistically significant relationship was found for log BAF_P and water concentration: log BAF_P= 1.97±0.11 – 0.49±0.08(Log conc;ng/L); R^2=0.51, P <0.001.

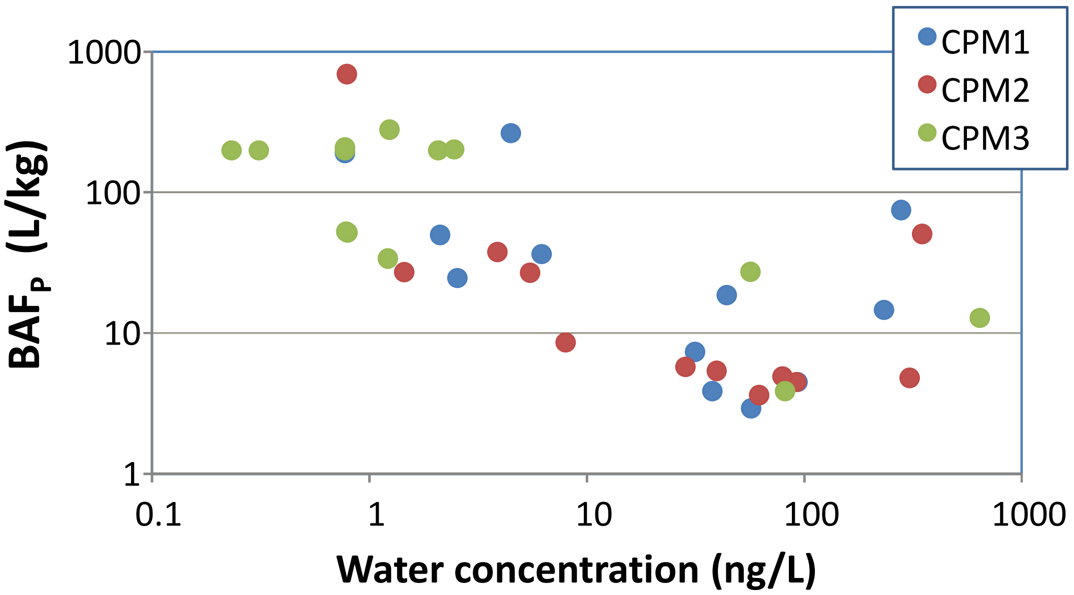

Figure S6. Measured BAF_P versus (A) predicted BAF_{WB} and (B) predicted blood-water partition coefficients (P_{BW}s) for PPCPs detectable in wild goldfish and carp from Cootes Paradise Marsh. Further details are given in Table S5. Omitting sertraline, measured log BAF_P was correlated with predicted logBAF_{BW} (r^2=0.32, P=0.0436, N=13) for goldfish but not for carp (r^2=0.18, P=0.150, N=13). Log P_{BW}s were not correlated with log BAF_Ps (P >0.05). The 1:1 line for measured BAF_P versus predicted BAF_{WB} and P_{BW} is shown with a dashed line. Abbreviations: ΣAmitriptyline=Amitr; Benztropine=Benz; Citalopram=Cital; ΣDiazepam = Diaz; N,N-Diethyl-m-toluamide=DEET; Erythromycin = Eryth; Diphenhydramine = Diphen; Flumequine=Flume, ΣFluoxetine=Fluox; Gemfibrozil=Gem; ΣIbuprofen=Ibupr; Iopamidol=Iopam; Sulfamethazine=Sulfam; Triclocarban=Triclo; Valsartan = Vals; Venlafaxine = Venla; Sertraline = Sertr.

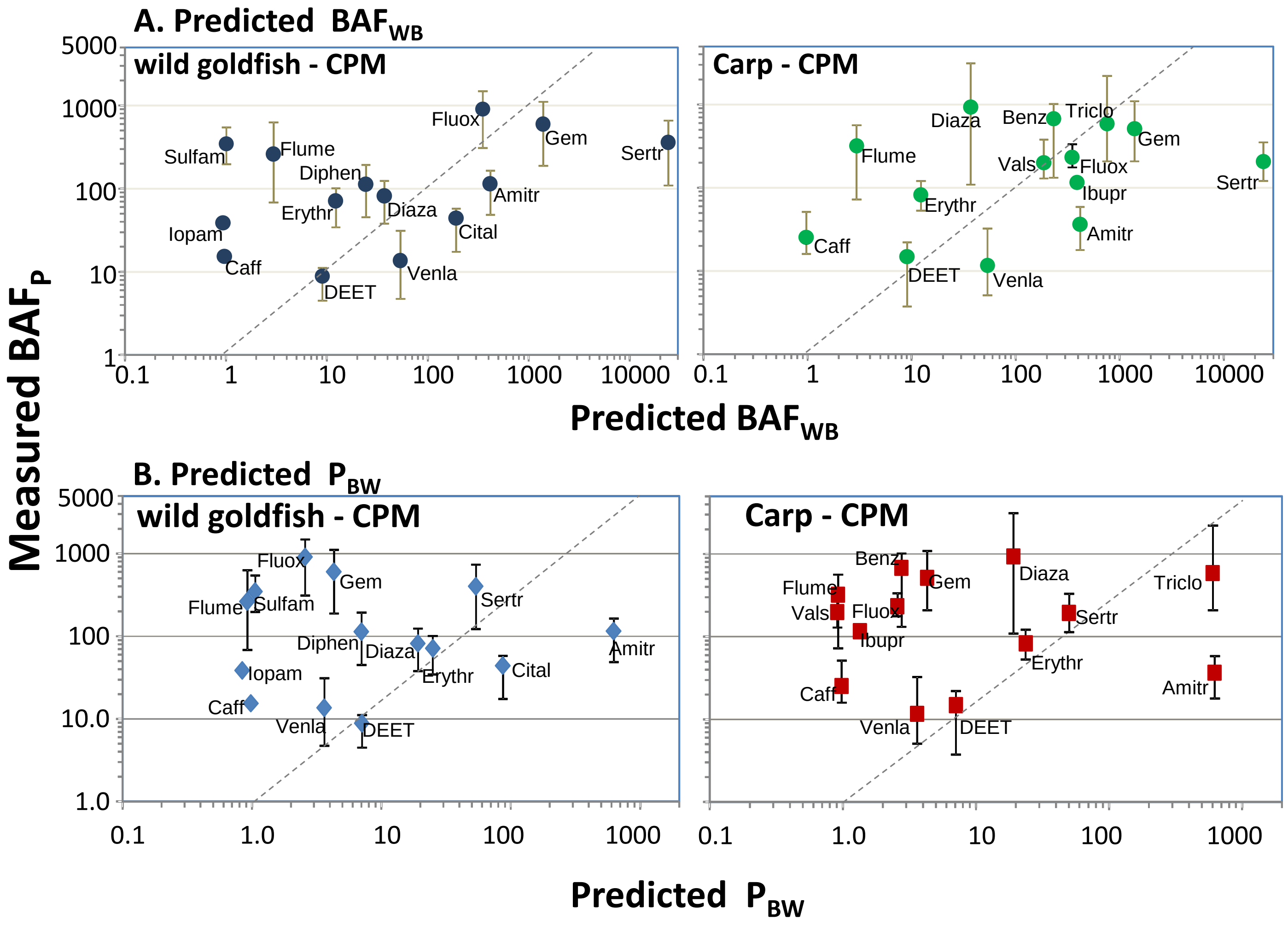

Figure S7. Measured BAF_P and predicted fish plasma concentrations (FPC) for PPCPs detectable in wild goldfish and carp from Cootes Paradise Marsh. The 1:1 line for measured versus predicted FPC is shown with a dashed line. Further details are given in Table S5.

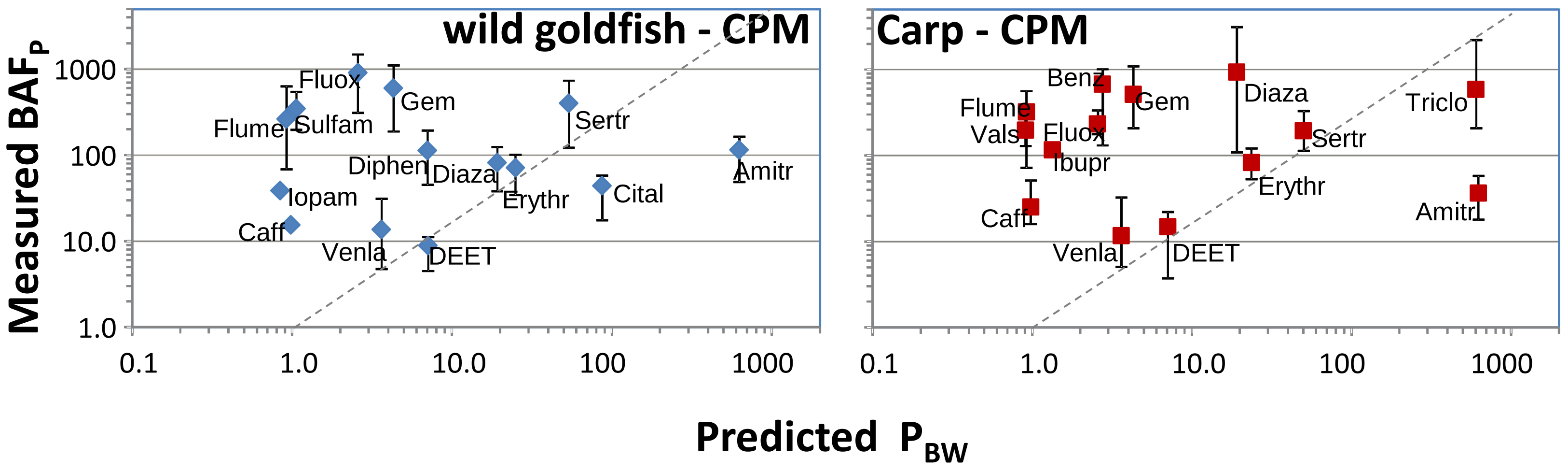

References cited in Supplementary Information

1 US EPA. Method 1694: Pharmaceuticals and Personal Care Products in Water, Soil, Sediment, and Biosolids by HPLC/MS/MS. EPA-821-R-08-002. (US Environmental Protection Agency, Office of Science and Technology, Engineering and Analysis Division (4303T), Washington, DC, 2007).

2 Lindholm-Lehto, P. C., Ahkola, H. S. J., Knuutinen, J. S. & Herve, S. H. Widespread occurrence and seasonal variation of pharmaceuticals in surface waters and municipal wastewater treatment plants in central Finland. *Environmental Science and Pollution Research* **23**, 7985-7997 (2016).

3 Golovko, O., Kumar, V., Fedorova, G., Randak, T. & Grabic, R. Seasonal changes in antibiotics, antidepressants/psychiatric drugs, antihistamines and lipid regulators in a wastewater treatment plant. *Chemosphere* **111**, 418-426 (2014).

4 Furlong, E. T., Noriega, M. C., Kanagy, C. J., Kanagy, L. K., Coffey, L. J. *et al.* Determination of human-use pharmaceuticals in filtered water by direct aqueous injection: high-performance liquid chromatography/tandem mass spectrometry. Report No. 5-B10, 60 (Reston, VA, 2014).

5 Simmons, D., Miller, J., Clarence, S., McCallum, E., Balshine, S. *et al.* Altered expression of metabolites and proteins in wild and caged fish exposed to wastewater effluents in situ. *Nature Scientific Reports, submitted* (2017).

6 Simmons, D. B. D., Bols, N. C., Duncker, B. P., McMaster, M., Miller, J. *et al.* Proteomic profiles of white sucker (catostomus commersonii) sampled from within the thunder bay area of concern reveal up-regulation of proteins associated with tumor formation and exposure to environmental estrogens. *Environmental Science and Technology* **46**, 1886-1894 (2012).

7 Berninger, J. P., Du, B., Connors, K. A., Eytcheson, S. A., Kolkmeier, M. A. *et al.* Effects of the antihistamine diphenhydramine on selected aquatic organisms. *Environ. Toxicol. Chem.* **30**, 2065-2072 (2011).

8 Huggett, D. B., Cook, J. C., Ericson, J. F. & Williams, R. T. A Theoretical Model for Utilizing Mammalian Pharmacology and Safety Data to Prioritize Potential Impacts of Human Pharmaceuticals to Fish. *Human and Ecological Risk Assessment: An International Journal* **9**, 1789-1799 (2003).

9 aus der Beek, T., Weber, F. A., Bergmann, A., Hickmann, S., Ebert, I. *et al.* Pharmaceuticals in the environment—Global occurrences and perspectives. *Environmental Toxicology and Chemistry* **35**, 823-835 (2016).

10 Huerta, B., Rodríguez-Mozaz, S. & Barceló, D. Pharmaceuticals in biota in the aquatic environment: Analytical methods and environmental implications. *Analytical and Bioanalytical Chemistry* **404**, 2611-2624 (2012).

11 Brown, J. N., Paxéus, N., Förlin, L. & Larsson, D. G. J. Variations in bioconcentration of human pharmaceuticals from sewage effluents into fish blood plasma. *Environmental Toxicology and Pharmacology* **24**, 267-274 (2007).

12 Fick, J., Lindberg, R. H., Parkkonen, J., Arvidsson, B., Tysklind, M. *et al.* Therapeutic levels of levonorgestrel detected in blood plasma of fish: results from screening rainbow trout exposed to treated sewage effluents. *Environmental Science and Technology* **44**, 2661-2666 (2010).

13 Du, B., Haddad, S. P., Luek, A., Scott, W. C., Saari, G. N. *et al.* Bioaccumulation and trophic dilution of human pharmaceuticals across trophic positions of an effluent-dependent wadeable stream. *Philosophical Transactions of the Royal Society B: Biological Sciences* **369** (2014).

14 Scott, W. C., Du, B., Haddad, S. P., Breed, C. S., Saari, G. N. *et al.* Predicted and observed therapeutic dose exceedances of ionizable pharmaceuticals in fish plasma from urban coastal systems. *Environ. Toxicol. Chem.* **35**, 983-995 (2016).

15 Zhao, J.-L., Liu, Y.-S., Liu, W.-R., Jiang, Y.-X., Su, H.-C. *et al.* Tissue-specific bioaccumulation of human and veterinary antibiotics in bile, plasma, liver and muscle tissues of wild fish from a highly urbanized region. *Environmental Pollution* **198**, 15-24 (2015).

16 Tanoue, R., Nomiyama, K., Nakamura, H., Kim, J.-W., Isobe, T. *et al.* Uptake and Tissue Distribution of Pharmaceuticals and Personal Care Products in Wild Fish from Treated-Wastewater-Impacted Streams. *Environmental Science & Technology* **49**, 11649-11658 (2015).

17 Moreno-González, R., Rodríguez-Mozaz, S., Huerta, B., Barceló, D. & León, V. M. Do pharmaceuticals bioaccumulate in marine molluscs and fish from a coastal lagoon? *Environmental Research* **146**, 282-298 (2016).

18 Meador, J. P., Yeh, A., Young, G. & Gallagher, E. P. Contaminants of emerging concern in a large temperate estuary. *Environmental Pollution* **213**, 254-267 (2016).

19 US EPA. Exposure Assessment Tools and Models, Estimation Program Interface (EPI) Suite Ver 4.1. (US Environmental Protection Agency, Office of Pollution Prevention and Toxics, Washington, DC, 2011).
